# Depletion of the RNA binding protein HNRNPD impairs homologous recombination by inhibiting DNA-end resection and inducing R-loop accumulation

**DOI:** 10.1093/nar/gkz076

**Published:** 2019-02-25

**Authors:** Luigi Alfano, Antonella Caporaso, Angela Altieri, Milena Dell’Aquila, Claudia Landi, Luca Bini, Francesca Pentimalli, Antonio Giordano

**Affiliations:** 1 Oncology Research Center of Mercogliano (CROM); Istituto Nazionale Tumori, IRCCS, Fondazione G. Pascale, Napoli, Italia; 2 Department of Medical Biotechnologies, University of Siena, Siena, Italia; 3 Sbarro Institute for Cancer Research and Molecular Medicine, Center for Biotechnology, College of Science and Technology, Temple University, Philadelphia, PA, USA; 4 Department of Life Sciences, University of Siena, Siena, Italia

## Abstract

DNA double strand break (DSB) repair through homologous recombination (HR) is crucial to maintain genome stability. DSB resection generates a single strand DNA intermediate, which is crucial for the HR process. We used a synthetic DNA structure, mimicking a resection intermediate, as a bait to identify proteins involved in this process. Among these, LC/MS analysis identified the RNA binding protein, HNRNPD. We found that HNRNPD binds chromatin, although this binding occurred independently of DNA damage. However, upon damage, HNRNPD re-localized to γH2Ax foci and its silencing impaired CHK1 S345 phosphorylation and the DNA end resection process. Indeed, HNRNPD silencing reduced: the ssDNA fraction upon camptothecin treatment; AsiSI-induced DSB resection; and RPA32 S4/8 phosphorylation. CRISPR/Cas9-mediated HNRNPD knockout impaired *in vitro* DNA resection and sensitized cells to camptothecin and olaparib treatment. We found that HNRNPD interacts with the heterogeneous nuclear ribonucleoprotein SAF-A previously associated with DNA damage repair. HNRNPD depletion resulted in an increased amount of RNA:DNA hybrids upon DNA damage. Both the expression of RNase H1 and RNA pol II inhibition recovered the ability to phosphorylate RPA32 S4/8 in HNRNPD knockout cells upon DNA damage, suggesting that RNA:DNA hybrid resolution likely rescues the defective DNA damage response of HNRNPD-depleted cells.

## INTRODUCTION

DNA double strand breaks (DSBs), are among the most potent genotoxic lesions, being able to induce chromosomal rearrangements (1) and therefore constituting a major challenge to genomic stability. DSBs can occur during physiological processes, such as DNA replication, recombination and lymphoid cell development, or can be induced by exogenous agents such as ionizing radiation (IR) and radiomimetic chemicals, including many anticancer drugs (2). Defects in genes involved in DSB repair have been associated with a wide range of diseases, from neurodegenerative disorders to syndromes with increased cancer risk and premature aging (3,4).

To safeguard genome stability and increase survival, cells use two principal pathways for DSBs repair: non-homologous end-joining (NHEJ) (5) and homologous recombination (HR) (6). The main difference between these two pathways consists in the fact that NHEJ, by joining DNA ends irrespectively of their original sequence, is error-prone, whereas HR restores the correct information using the sister chromatid as a faithful template. While NHEJ can function throughout the cell cycle, HR is restricted to late S and G2 phases (7) when sister chromatids are available (5,6). A necessary step for HR is the generation of long 3′ single-stranded DNA (ssDNA), obtained through the DNA end-resection process, which is triggered by the recruitment onto the DNA lesions of the MRN complex (MRE11–RAD50–NBSI) and CTIP (RBBP8), which stimulates MRE11 activity (8,9). MRE11, which is endowed of both endo and exonuclease activity, promotes the formation of minimally resected ends by nicking DNA in multiple positions flanking the breaks, acting in concert with the recently identified EXD2 exonuclease (10). Following initial resection the EXOI nuclease and the DNA2 helicase, in complex with the Bloom syndrome helicase (BLM) (11), further process the breaks generating longer ssDNA tails, which are bound by the RPA complex to prevent hairpin formation (12) and to facilitate the loading of RAD51 for the strand exchange process (13). SsDNA, generated both at the replication fork or during the DNA resection process, is a unstable structure which is exposed to the possible hybridization with the nascent RNA to form DNA:RNA hybrids (R-loops) (14). Emerging evidences showed that proper processing of R-loops during DNA repair is required to preserve genome integrity (14). In particular, R-loop resolution driven by the DDX1 RNA helicase was found to be essential for the HR process in human cells and, similarly, in yeast cells in which RNase H activity is required for the RPA recruitment during HR (15,16).

Here, through a proteomic screening, using a synthetic DNA mimicking a DNA-end resection intermediate, we identified the mRNA binding protein HNRNPD (heterogeneous nuclear ribonucleoprotein D), as a novel player in the resection process, which favours the DNA:RNA hybrid removal for a proper HR resolution.

## MATERIALS AND METHODS

### Cell culture, DNA constructs and transfection

The HeLa cell line was obtained by the American Type Culture Collection (ATCC, CCL-2, Manassas, VA, RRID:CVCL_0030). Cell lines were cultured in RPMI 1640 (HeLa cells) (Thermo Fisher Scientific, Monza MB, IT) supplemented with 10% fetal bovine serum (Thermo Fisher Scientific), penicillin (100 U/ml), streptomycin (100 μg/ml) and 2 mM glutamine at 37°C in 5% CO_2_. The plasmids encoding the sequences of the HNRNPD isoforms (p45, p42, p40 and p37) fused to the FLAG-tag were a gift from R.J. Schneider, Department of Microbiology and Radiation Oncology, NYU School of Medicine. The plasmid encoding SAF-A-FLAG wt was a gift from Nick Gilbert, MRC Human Genetics Unit, Institute of Genetics and Molecular Medicine, University of Edinburgh, Crewe Road, Edinburgh, UK. The plasmid encoding the human GFP-RNase H1 was a gift from Robert Joseph Crouch, Developmental Biology Division, Eunice Kennedy Shriver National Institute of Child Health and Human Development, National Institutes of Health, Bethesda, MD, USA. To generate the HNRNPD mutants we cloned into the pFLAG CMV-1 vector, through EcoRI and BamHI sites, the corresponding DNAs amplified by PCR through the primers listed in Supplementary Table S1. To generate the HNRNPD mutants for *E. coli* expression we cloned in pET-duet vector, through the EcoRI and HindIII sites, the corresponding DNAs amplified by PCR through the primers listed in Supplementary Table S1. To generate HeLa cell lines stably expressing ER-AsiSI (17), cells were seeded at 90% confluence on 6-well plates and transfected with 1μg of pBABE ER-AsiSI (a gift from G. Legube, Center for Integrative Biology, Université Paul Sabatier, France) through Lipofectamine 2000 Reagent (Thermo Fisher Scientific). ER AsiSI-expressing cells were selected using puromycin (Sigma Aldrich S.r.l, Milan IT) at the previously optimized concentration of 5 μg/ml. For silencing experiments, HeLa cells were transfected with, ON-TARGETplus Human HNRNPD siRNA Dharmacon, 30 nM of siCTR (D-001810–10) or siHNRNPD (L-004079) using Dharmafect 1 according to the manufacturer instructions. ON-TARGETplus siRNA are optimized to achieve high reduction of off target effects. The HeLa HNRNPD knockout cells were generated through the CRISPR-Cas9 system. Briefly, cells were transfected with the pSpCas9 (BB)-2A-Puro (PX459) V2.0 (18) (gift from Feng Zhang, Addgene plasmid #62988) containing guide RNAs targeting the HNRNPD exon two (5′-TCCTATCACAGGGCGATCAA-3′) and selected with 5μg/ml of puromycin. The generated cell clones were analyzed by western blot and sequencing to verify knockout of HNRNPD. For reconstitution experiments we generated the PAM resistant HNRNPD isoforms through the QuikChange II Site-Directed Mutagenesis Kit (Agilent) according to the manufacturer instructions with the primers indicated in Supplementary Table S1.

The pCBASceI and pDRGFP plasmids were a gift from Maria Jasin, Addgene plasmid #26477 (19) and Addgene plasmid #26475 (20), respectively.

### Antibodies and western blot

The following antibodies were used: HNRNPD (1:1000, 07-260, Millipore, RRID:AB_2117338), HNRNPD (1:1000, D6O4F, Cell Signalling, Danvers, MA, RRID:AB_2616009), RPA32 (1:5000, A300–244A, Bethyl Laboratories, RRID:AB_185548), RPA32 S4/S8 (1:2000, A300–245A, Bethyl Laboratories), H3 (1:1000, #9715, Cell Signalling, RRID:AB_331563), CHK1 S345 (1:1000, #2348, Cell Signalling), CHK1 (1:1000, #2360, Cell Signalling), GAPDH (1:1000, sc-25778, Santa Cruz, Dallas), MRE11 (1:1000, NB100–142, Novus Biological), EXOI (1:1000, A302–640A, Bethyl Laboratories), CtIP (1:1000, #61142, Active Motif) SAF-A (1:1000, ab10297, Abcam), RAD17 S645 (1:2000, ab3620, Abcam), FLAG-M2 (1:1000, F1804, SIGMA Aldrich), HA-tag (1:500, sc-805, Santa Cruz Biotechnology), Lamin A/C (1:1000, #4777, Cell Signalling), GFP (1:5000, ab6556, Abcam), His-tag (1:1000, 05-531, Millipore). For total protein extraction, cells were lysed at 4°C in 50 mM HEPES pH7.5, 1% Triton X-100, 150 mM NaCl, 5 mM EGTA, supplemented with protease and phosphatase inhibitor cocktail (Roche Applied Science). Lysates were clarified by centrifugation at 10 000 × g for 20 min. Lysates containing equal amounts of proteins, estimated through the Bradford assay (Bio-Rad), were subjected to SDS-page. The chemiluminescent images were obtained using the ImageQuant LAS 500 (GE Healthcare).

### Immunoprecipitation

For protein co-immunoprecipitation, HeLa cells were lysed in protein extraction buffer as for western blot. The protein lysate was quantified and 2 mg, for each condition, were pre-cleared with protein G plus agarose (22851, Thermo Fischer Scientific) 45 min at 4°C on rocking. Immunoprecipitation was carried out at 4°C on rocking over night with either FLAG-M2 (1 μg Ab to 1 mg of proteins, F1804, Sigma Aldrich) and its negative control IgG1 (BD Pharmingen™), or HA-tag (5 μg Ab to 2 mg of proteins, 000000011583816001, Sigma Aldrich) and its negative control IgG2 (550339, BD Pharmingen™).

### RPA-ssDNA/dsDNA pull-down

**Figure 1.**
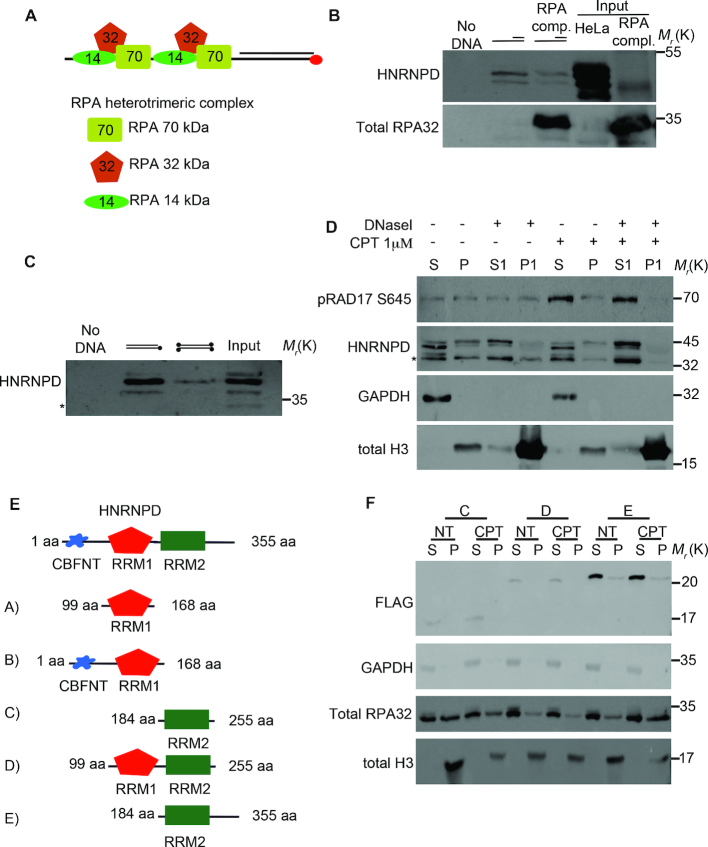
Proteomic screen and HNRNPD chromatin binding ability. (**A**) Schematic representation of the proteomic screen by using a synthetic DNA structure coated with the heterotrimeric RPA^wt^ complex, including the 70, 32 and 14 kDa isoforms, which was used as bait for the proteomic screen after being challenged with HeLa nuclear extracts. (**B**) DNA pull down assay of the synthetic DNA structure coated or not with the recombinant RPA complex produced in *E. coli* (input) followed by western blot analysis with the indicated antibodies. HeLa nuclear extract was incubated for 30 min with 0,3 mg/ml of RNase A on ice, followed by centrifugation to remove debris. (Sequences are listed in Supplementary Table S1). (**C**) DNA pull-down of the schematically indicated biotinylated DNA structures (Sequences are listed in Supplementary Table S1). Biotin is represented as black dots. Western blot analysis was performed with the indicated antibodies. The asterisk indicates a non-specific band (which appears using the Millipore antibody). (**D**) Chromatin enriched purification was performed upon 2 h treatment with 1μM of CPT followed by western blot analysis. DNase I treatment of the first pellet fraction (P) was performed, when indicated, with 80U of enzyme for 30 min at 30°C originating a new supernatant and pellet fraction (S1 and P1 respectively). All the purification steps were performed upon incubation for 30 min with 0,3 mg/ml of RNase A on ice, followed by centrifugation to remove debris. H3 and GAPDH were used as markers for the chromatin and soluble fractions, respectively. RAD17 S645 was used as a DNA damage control. (**E**) Schematic representation of HNRNPD deletion mutants. (**F**) HeLa cells were transfected with the indicated DNA, followed by 48 h of incubation. Chromatin enriched purification was performed upon 2 h treatment with 1μM of CPT followed by western blot analysis. All the purification steps were performed upon incubation for 30 min with 0.3 mg/ml of RNase A on ice, followed by centrifugation to remove debris. H3 and GAPDH were used as markers for the chromatin and soluble fractions, respectively. RPA32 was used as a DNA damage control.

Biotinylated DNA pull-down assay was performed as reported by Yang and Zou (21) with some modifications (see Figure 1). Briefly, 87 pmol of 70 nt biotinylated ssDNA were annealed with 87 pmol of 21 nt ssDNA, partially complementary, to generate the DNA end-resection intermediate used as bait in the proteomic screening, or with the same amount of other, different length, ssDNAs to generate DNA fragments either blunt or with varying ssDNA length. Reactions were performed in annealing buffer (20mM NaCl, 10mM Tris–HCl pH7.5) for 3 min at 90°C followed by incubation 15 min at 37°C in a water bath. Annealed DNA was attached to streptavidin-coated magnetic beads (Thermo Fisher Scientific) in binding buffer (10 mM Tris–HCl pH 7.5, 100 mM NaCl, 10% glycerol, 0.01% NP-40) for 15 min rocking at RT followed by incubation with or without purified RPA at RT for 30 min. At the end of incubation, DNA-streptavidin-coated magnetic bead complexes were washed twice with the binding buffer to remove the unbound proteins and subsequently incubated with protein extract from HeLa enriched for the nuclear fraction at RT for 30 min, followed by two washes before western blot analysis or LC/MS. The DNA sequences used are listed in Supplementary Table S1.

### Cell fractionation

Cell fractionation was performed as previously described by Ishii *et al.* with minor modifications (22). Briefly, 3 × 10^6^ cells, per condition, were collected and resuspended in 200 μl of CSK buffer (10 mM PIPES pH 6.8, 100 mM NaCl, 300 mM MgCl_2_, 1 mM EGTA, 1 mM DTT, 0.1% Triton X-100, 0.34 M sucrose) supplemented with protease and phosphatase inhibitors and kept 5 min on ice. The soluble cytoplasmic fraction (S) was separated from nuclei (P) by 4 min centrifugation at 1300 × g at 4°C. The P fraction was washed with CSK then resuspended in 200 μl of ‘western blot buffer’, sonicated and centrifuged for 30 min at 4°C at 10 000 × g. Following SDS-PAGE, samples were analyzed by western blot with the indicated antibodies. For DNase I treatment the P fraction was incubated with 80U of DNase I (Roche Applied Science, Mannheim Germany) for 30 min at 30°C followed by 30min centrifugation at 1300 × g at 4°C to obtain the solubilized fraction (S1) and pellet post DNase A (P1).

### Immunofluorescence and UVC micro-irradiation

HeLa cells, grown on glass coverslips, were fixed with 4% paraformaldehyde and permeabilized with 0.5% triton. Samples were blocked 10 min in 1%BSA at RT and incubated 1 h with anti-BrdU (1:200, 347580, BD Biosciences) or anti-γH2Ax S139 (1:600, 05-636, Millipore) 37°C. After washing, samples were incubated 45 min at 37°C with AlexaFluor 594-conjugated chicken anti-rabbit and 488-conjugated rabbit anti-mouse or 555-conjugated goat anti-mouse IgG (H+L) (Life Technologies), and analyzed with a Zeiss LSM100 confocal microscope.

UVC micro-irradiation was performed as described by Suzuki *et al.* (23), then the following antibodies were used for protein detection HNRNPD (1:200, 07-260, Millipore), γH2Ax S139 (1:600, 05-636, Millipore).

### Flow cytometry analysis

Upon 48 h post transfection with siRNA, HeLa cells were treated or not with 1 μM camptothecin (CPT) (Sigma Aldrich) for 2 h and processed as reported by Forment *et al.* (24) with some modifications. Briefly, 1 × 10^6^ HeLa cells, per condition, were resuspended in CSK buffer + 0.5% Triton + 0.3 mg/ml RNase A solution with protease inhibitors and incubated for 5 min on ice. At the end of incubation cells were washed twice with CSK buffer followed by centrifugation for 3 min a 1300 × g at 4°C. Afterwards, cells were fixed with 4% paraformaldehyde for 15 min at RT, washed twice in PBS1X and re-suspended in 100 μl of incubation buffer (PBS 1× + 0.5% Saponin) for 1 h with, RPA (1:200, A300-244A, Bethyl Laboratories) or γH2Ax S139 (1:200, 05-636, Millipore) at 37°C. After two washes with incubation buffer, samples were incubated 45 min at 37°C with Alexa Fluor 594-conjugated chicken anti-rabbit or 488-conjugated rabbit anti-mouse (Thermo Fisher Scientific). The percentage of positive cells was determined using the CellQuest Software (Becton Dickinson).

The ssDNA formation assay was performed as reported previously (25) with some modifications. Briefly, HeLa cells, 24 h after transfection with the indicated siRNAs, were pulse-labelled for additional 24 h with 10 μM BrdU and treated with 1 μM CPT for 2 h. In order to quantify the amount of resected ssDNA we worked in non-denaturing conditions. Cells were harvested by trypsinization. After washing with the PBS1X, 1 × 10^6^ cells for each experimental point were fixed in PBS 1× + 4% paraformaldehyde for 15 min at RT followed by permeabilization with PBS 1× containing 0.1% Triton X-100 for 30 min at RT. Cells were washed in PBS 1× twice and re-suspended in 100μl of incubation buffer (PBS 1× + 0.5% Saponin) with the anti-BrdU antibody (1:100, clone B44, 347580, BD Biosciences) for 1 h at RT or just incubation buffer as control. Cells were washed with incubation buffer and resuspended with 100 μl of 488-conjugated rabbit anti-mouse (Thermo Fisher Scientific) for 45 min at RT. The percentage of BrdU positive cells was determined using the CellQuest Software (Becton Dickinson). The threshold level identifying FITC positivity was set following comparison with cells incubated with only the secondary antibody.

### Nuclear extract preparation

Nuclear extracts were prepared as previously reported (26). The nuclear fraction was dialyzed over night at 4°C in 10 mM Tris–HCl pH 7.5, 100 mM NaCl, 10% glycerol, 0.01% NP-40.

### SMART

SMART (single molecule analysis of resection tracks) was carried out as previously described by Cruz-Garcia *et al.* (27). The same number of control and HNRNPD silenced cells was spotted onto the slides, so, presumably, the same DNA content attached to the slides, although we have not assessed this. However the SMART technique was used to gain a snapshot visual assessment of the effect of HNRNPD silencing on bulk ssDNA amount upon CPT.

### ER-AsiSI resection assay

Genomic DNA extraction and preparation for the measurement of resection in mammalian cells was performed as previously (28) described with some modifications. Briefly, HeLa cells, stably expressing the ER-AsiSI system (gift from Gaelle Legube CBI-Centre de Biologie Integrative-Toulouse), were treated with or without 300 nM of 4-OHT (Sigma Aldrich) for 4 h. Cells were then trypsinized and resuspended in 0.6% Low gelling agarose (Sigma Aldrich) at a concentration of 6 × 10^6^ cells/ml. Fifty microliters of cells were spotted onto a piece of Parafilm to produce an agar solid ball, which was then resuspended in 1 ml of ESP buffer (0.5 M EDTA, 2% *N*-lauroylsarcosine, 1 mg/ml proteinase-K, 1 mM CaCl_2_, pH 8.0) for 20 h at 16°C with rotation followed by treatment with 1 ml of HS buffer (1.85 M NaCl, 0.15 M KCl, 5 mM MgCl_2_, 2 mM EDTA, 4 mM Tris, 0.5% Triton X-100, pH 7.5) for 20 h at 16°C with rotation. After 6X washes of 10 min each with ice cold PBS1X (8 mM Na_2_HPO_4_, 1.5 mM KH_2_PO_4_, 133 mM KCl, 0.8 mM MgCl_2_, pH 7.4) at 4°C, the agar ball was melted at 70°C for 15 min and then diluted 15-fold with 70°C of ddH_2_O. The DNA was diluted with an equal volume of 2X NEB 3.1 buffer. Twenty microliters of genomic DNA were digested or mock digested with 20 units of BamHI-HF (NEB) over night at 37°C. Four microliters were used as template for the real time quantitative PCR reaction (qRT-PCR) with the indicated primers using the SYBR Green real time master mix (Thermo Fisher Scientific). All qRT-PCR reactions were performed in a 7900HT fast-RealTimePCR (Applied Biosystem). The ΔCt value was calculated by subtracting the Ct value of the mock-digested sample from the Ct value of the digested sample. The %ssDNA = 1/(2^(ΔCt-1)^+0.5) × 100 (28). The sequences of primers used are listed in Supplementary Table S1.

### Chromatin immunoprecipitation (ChIP)

ChIP was carried out as reported by Lee *et al.* (29) with some modifications. HeLa cells expressing ER-AsiSI were mock-treated or treated with 300 nM of 4-OHT for 1 h. Cells were cross-linked with 1% formaldehyde for 10 min at 37°C followed by inactivation with 0.125 M glycine. Cells were washed twice with ice cold PBS 1X; for each condition 1 × 10^6^ cells were re-suspended in ChIP lysis buffer (50 mM Tris–HCl pH 8, 10 mM EDTA pH 8, SDS 0.1% and protease inhibitors) and incubated 10 min on ice followed by sonication (20 s-on/10 s-off twelve times at 90% of amplitude using Sonics Vibra-Cell Sonics, Newtown, CT, USA). Lysate was clarified for 30 min at 14 000 rpm at 4°C and diluted tenfold with the dilution buffer (50 mM Tris–HCl pH 8, 150 mM NaCl, 1% Triton X-100, SDS 0.1% and protease inhibitors). FLAG M2 antibody (Sigma Aldrich) was used to immunoprecipitate the HNRNPD p45-FLAG or SAF-A-FLAG wt over night at 4°C. To isolate the immunocomplexes, 20 μl of protein G plus agarose (Thermo Fisher Scientific) was added to each sample and incubated, on rocking, 45 min at 4°C. The pellets were sequentially washed with low salt buffer (0.1% SDS, 1% Triton X-100, 2 mM EDTA pH8, 20 mM Tris–HCl pH8, 50 mM NaCl), high salt buffer (0.1% SDS, 1% Triton X-100, 2 mM EDTA pH 8, 20 mM Tris–HCl pH 8, 500 mM NaCl), LiCl buffer (0.25 M LiCl, 1% NP40, 1 mM EDTA pH 8, 1% deoxycholate acid, 10 mM Tris–HCl pH 8) and TE buffer twice (10 mM Tris–HCl ph8 and 1mM EDTA) followed by cross-link reversion in elution buffer (1% SDS and 0.1 M NaHCO_3_) with 270 mM NaCl at 65°C for 4 h. Protein digestion was carried out by adding 12 mM EDTA pH 8, 54 mM Tris–HCl pH 8 and 30 μg of proteinase K for 1 h at 45°C. DNA was recovered by phenol/chloroform extraction and analyzed, with the indicated primers, using a SYBR Green real time master mix (Thermo Fisher Scientific). All qRT-PCR reactions were performed in a 7900HT fast-RealTimePCR (Applied Biosystems). Primers sequences used as qRT-PCR are listed in Supplementary Table S1 (28). Data are reported as mean ± s.d. of three independent experiments. IP efficiency was calculated as % of immunoprecipitated input DNA.

### Cell cycle profile

For DNA content analysis cells were fixed in ice-cold 70% ethanol at –20°C. At least 10 000 cells were analyzed by FACS (Becton Dickinson) following staining with 5 mg/ml propidium iodide and 0.25 mg/ml RNase A treatment (Sigma-Aldrich). Data were analyzed through the CellQuest Software (Becton Dickinson).

### Quantitative real time PCR

Total RNA was extracted using Trizol (Life Technologies) and treated with TurboDNase (Life Technologies). 1 μg of RNA was retro-transcribed using the Superscript VILO cDNA synthesis kit (Life Technologies). cDNA samples were amplified by real-time quantitative reverse transcriptase-PCR (qRT-PCR) using SYBR Green PCR Master Mix (Life Technologies) with the primers listed in Supplementary Table S1. Expression levels were normalized to those of the β-actin gene. *HNRNPD, MRE11, CTIP* and *EXOI* expression levels in siHNRNPD cells were calculated by the 2^–ΔΔCt^ method relatively to siCTR control cells.

### Colony formation assay

For clonogenic assays, 300 cells were seeded in 24-wells plates and either untreated or treated with the indicated doses of CPT or olaparib (Selleckem) and incubated for 10 days. Colonies were counted after fixation with methanol and staining with crystal violet.

### In vitro DNA end-resection assay

Nuclear proteins from HeLa wt and HNRNPD KO cl10 were purified as described above for nuclear extract preparation, followed by dialysis over night at 4°C in 50 mM Tris–HCl pH 7.5, 50 mM NaCl, 2 mM MgCl_2_, 1 mM DTT and 0.1 mg/ml BSA. A DNA plasmid vector was digested with KpnI (5′ overhangs), HindIII (3′ overhangs) or EcoRV (blunt ends) followed by column purification (Qiagen, Germantown, MD, USA). The reactions were carried out in a final volume of 20 μl with 5 μg of nuclear proteins, per condition, and 300 ng of linearized DNA vector for the indicated time points followed by incubation in 10 mM EDTA, 0.25% SDS and 100 μg/ml proteinase K for 10 min at 37**°**C. DNA products separated on 0.8% agarose were stained with ethidium bromide.

### DNA–RNA immunoprecipitation (DRIP)

DNA–RNA immunoprecipitation was carried out as reported by Li *et al.* (15) with some modifications. HeLa ER-AsiSI were transfected with siCTR or siHNRNPD for 48 h followed by treatment with 4-OHT 300 nM for 4 h. For each condition, 5 × 10^6^ cells were re-suspended in TE buffer (10 mM Tris–HCl pH 7.5 + 0.5 mM EDTA) + 0.5% SDS + 300 μg/ml proteinase K a 37°C over night under agitation. At the end of incubation, the DNA:RNA hybrids were extracted with the phenol/chlorophorm protocol; the precipitate was washed with 70% of EtOH and air dried. The pellet was resuspendend in 100 μl of H_2_O and digested over night with 50 U of each of the following restriction enzymes (BsaI, BstXI, NdeI, EcoRI, EcoRV) in 1X restriction buffer. DNA:RNA hybrids were purified through phenol/chlorophorm. The pellet was resuspended in 50 μl of water and treated or mock treated with 15 U di RNase H (18021071, Thermo Fischer Scientific) in a final volume of 100 μl and incubated over night at 37°C. DNA:RNA hybrids were purified through phenol/chlorophorm and resuspended in 50 μl of H_2_O. 5μg of digested DNA were incubated with 10 μg of S9.6 antibody (ENH001, Kerafast) in binding buffer (10 mM Tris–HCl ph 7.5, 1 mM EDTA, 10 mM NaPO_4_, 140 mM NaCl, 0.05% Triton X-100) over night a 4°C. At the end of incubation, we added 20 μl of protein G plus agarose (22851, Thermo Fischer Scientific) for 2 h at 4°C on rocking; washed three times with binding buffer and elution of immunocomplexes with five times of protein G volume with 50 mM Tris–HCl pH 7.5, 10 mM EDTA, 0.5% SDS, 500 μg/ml proteinase K for 45 min at 55°C. DNA:RNA hybrids were purified with the phenol/chlorophorm protocol and resuspended in 50 μl of H_2_O. Four microliters were used as template for the real time quantitative PCR reaction (qRT-PCR) with primers amplifying a region which is ∼600 bp from the AsiSI-induced DSB site (listed in Supplementary Table S1 DSB-F/R ChIP and DRIP) using the SYBR Green real time master mix (Thermo Fisher Scientific).

### RPA complex cloning and purification

Molecular cloning of the wt RPA complex was performed in pET-duet vector with the primers listed in the Supplementary Table S1. Purification of wt RPA was performed as reported previously (30). Briefly, wt RPA was transformed in BL21 DE3 (Rosetta) followed by induction with 300 μM IPTG for 4 h. The bacterial pellet was resuspended in lysis buffer (50 mM NaH_2_PO_4_ pH 7, 300 mM NaCl, 15 mM Imidazole and 10% glycerol followed by sonication). The Ni-NTA resin was used for affinity purification for 2 h at 4°C. At the end of incubation time, the resin was washed six times with 10 mM Tris–HCl pH 8, 300 mM NaCl and scalar concentration of imidazole from 10 mM to 60 mM. The RPA complex was eluted with 10 mM Tris–HCl pH 8, 300 mM NaCl and 300 mM imidazole. The dialysis was carried out over night at 4°C with 100 mM NaCl, 10 mM Tris–HCl pH 7.5, 10% glycerol and 0.01% NP40.

### Homologous recombination reporter assay

HeLa cells stable expressing the reporter plasmid pDR-GFP, were transfected with the pDR-GFP plasmid (20) (gift from Maria Jasin, Addgene plasmid #26475) and selected with puromycin. HeLa pDRGFP cell lines were co-transfected with the coding plasmid for the endonuclease I-SceI (pCBA SceI, a gift from Maria Jasin, Addgene plasmid #26477(19) and the siCTR, siHNRNPD or siMRE11 (L-009271, Dharmacon). Upon 48 h of incubation we analyzed the GFP values (as a readout of HR frequency) through the FACS analysis.

### Identification by LC–MS/MS

Peptide sequencing was performed on a Nano-scale by LC–ESI/MS–MS (31). LC–MS system consists of PHOENIX 40 (ThermoQuest Ltd., Hemel Hempstead, UK) connected to LCQ DECA Ion-Trap mass spectrometer (Finnigan, San Jose, CA, USA). Twenty microliters of trypsin-digested solutions were injected in a six-port valve and were trapped in a C18 trapping column (20 mm × 100 μm ID × 360 μm OD, Nano-separations, Nieuwkoop, NL) using 100% HPLC grade water + 0.1% v/v formic acid (solvent A) at a flow rate of 5 μl/min for 10 min. A pre-column splitter restrictor enabled the flow rate to be set at 100–125 nl/min on a C18 analytical column (30 cm × 50 μm ID × 360 μm OD, Nano-separations). Analytical separation was performed using a linear gradient up to 60% acetonitrile + 0.1% (v/v) formic acid (solvent B) for 60 min. At the end of separation, trapping and analytical columns were washed for 10 min in 100% solvent B and were equilibrated for 10 min in 100% solvent A. An ESI needle, composed of gold-coated fused silica (5 cm × 25 μm ID × 360 μm OD, Nano-separations), was heated to 195°C and 2 kV was applied for stable spray operation. Xcalibur™ 1.2 software (Thermo) managed the LC pump and the automatic spectral recording. MS/MS ion search was performed in Swiss-Prot/UniprotKB databases using MASCOT. We set *Homo sapiens* as taxonomy, peptide precursor charge at 2+ or 3+, mass tolerance at ±1.2 Da for precursor peptide and ±0.6 Da for fragment peptides, only one missed cleavage site as acceptable, carbamidomethylation of cysteine as fixed modification, and methionine oxidation as possible modification. Peptides with individual ion scores –10*log[P] were considered significant. The mass spectrometry proteomics data have been deposited to the ProteomeXchange Consortium via the PRIDE (32) partner repository with the dataset identifier PXD012045 and 10.6019/PXD012045.

### Statistical analysis and reproducibility

Paired two-sided Student's *t* test was used to compare the means of two matched groups; *P* < 0.05 was considered statistically significant. Representative experiments are shown out of at least two independent ones; detailed information (number of independent experiments, *P*-values) are listed in the individual figure legends.

## RESULTS

### Proteomic screen for the identification of new proteins involved in DNA end-resection

Recently, the ubiquitin ligase complex, PRP19, was identified as a novel player in the DNA damage response (DDR) by using biotinylated ssDNA in complex with RPA as a bait to identify binding proteins that could favour ATR activation (33). Here, we created a modified DNA structure in order to discover new proteins involved in the DNA end-resection process. To this aim, we annealed two different length DNA oligos, one of which was biotin-conjugated, forming a 21 bp dsDNA with a 49 nt protruding end, complexed with the *in vitro* purified wild type RPA heterotrimeric complex (34,35) (Figure 1A). Such structure, which mimics a DNA end-resection intermediate, was challenged with a nuclear-enriched protein extract from HeLa cells followed by a streptavidin pull-down assay. Bound proteins were identified upon tryptic digestion through mass spectrometry. From the analysis of the peptides associated with the RPA–DNA complex, beyond the RPA complex, three proteins were identified having significant Mascot scores in the Swiss-Prot/Uniprot KB database (Supplementary Figure 1a): X-ray repair cross-complementing protein 6 (XRCC6), also known as KU70, a key player of NHEJ, which rapidly binds DSBs (36); heterogeneous nuclear ribonucleoprotein A 1 (hnRNPA1), involved in various aspects of RNA metabolism and known to interact with telomeric DNA to regulate telomere length (37,38); and HNRNPD, also known as AUF1, a key factor in the regulation of mRNAs involved in proliferation, senescence and stress response, which consists of four different isoforms (p37, p40, p42, p45) deriving from alternative splicing (39).

### HNRNPD binds the chromatin DNA

HNRNPD was recently described to regulate, at the mRNA level, genes involved in the DDR thereby preserving genome stability through its activity on mRNAs (40). So, we set out to study the direct involvement of HNRNPD in the DDR. First, we verified HNRNPD ability to bind the synthetic DNA structure used as bait. Upon DNA pull-down and western blot, we found that HNRNPD is indeed able to bind to the synthetic DNA structure regardless whether the DNA was pre-coated with the RPA complex (Figure 1B). To test HNRNPD ability to bind the synthetic DNA sequence or its extremities, we used a reverse complementary synthetic DNA with either free or biotin-blocked ds-ends and found that HNRNPD binding was impaired by DNA-end blocking suggesting a possible role in the recognition of the DSB itself (Figure 1C). To test whether HNRNPD effectively binds cellular DNA, we performed chromatin purification, in the presence of RNase A, from HeLa cells treated with the topoisomerase I inhibitor camptothecin (CPT) (41) followed by western blot. CPT is a S-phase specific drug that inhibits the re-joining step of cleavage/religation upon topoisomerase I action (42), inducing the collapse of the replication fork and subsequent HR activation (43). We found that HNRNPD is able to bind chromatin. Indeed, the treatment of the chromatin-containing pellet fraction (P1) with DNase I actively releases HNRNPD (S1), although the binding is independent from the DNA damage stimulus (Figure 1D).

HNRNPD is an RNA binding protein with two RRM motifs conferring the binding activity to the mRNAs (44) and to the human single strand telomeric repeats (45). To assess whether the DNA binding ability of HNRNPD relies on the RRM motifs or other domains, we generated HNRNPD deletion mutants (Figure 1E) and tested their ability to bind chromatin upon enriched purification from HeLa cells treated or not with CPT; all the purification steps were carried out following treatment with RNase A. We found that only the mutant protein (E) containing the whole 100aa C-terminal portion was able to bind the chromatin DNA; whereas the mutants containing either one (C), or both RRM motifs (D), without the C-terminal region, were not (Figure 1F and data not shown for mutants A and B). To confirm that the loss of binding ability of mutants C and D was indeed due to their intrinsic nature and not to their lower expression, which could be below the technique detection threshold, we performed western blot analysis of the soluble and insoluble fraction using higher amounts of protein extracts, confirming the DNA binding defects of both mutants (Supplementary Figure 1b). Then, we tested the ability of HNRNPD mutants, transfected in HeLa cells, to bind the synthetic biotinylated DNA structure through streptavidin pull-down. Protein lysates were treated with RNase A to remove possible interferences by RNA molecules. As reported in supplementary Figure 1c, both endogenous and overexpressed HNRNPD wt proteins were able to bind the synthetic DNA. Consistent with the chromatin binding ability, the RRM2+C-terminal (E) deletion mutant preserves the DNA binding ability, contrary to the RRM1+RRM2 (D) mutant (Supplementary Figure 1c). Finally, we purified human recombinant His-tagged RRM1+RRM2 (D) or RRM2+C-terminal (E) mutant proteins expressed in *E. coli* to reduce the chance of pulling down interacting proteins; a streptavidin pull-down assay with the biotinylated DNA structure, confirmed that the E RRM2+C-terminal mutant retained its binding capacity whereas the D mutant did not (Supplementary Figure 1d). We could not test the C-terminal only because of difficulties in achieving its expression. Collectively, these data showed that HNRNPD is able to bind both DNA synthetic structures as well as chromatin from HeLa cells, independently of RNA; the DNA binding activity of HNRNPD likely requires the free DNA ends and the C-terminal protein domain, which, interestingly, contains a glycine–arginine rich domain (GAR) (46), which may favour its localization to the DSBs, as discussed below. Protein loading onto chromatin does not change in presence of DNA damage suggesting a possible protein re-localization upon DNA breaks.

### HNRNPD localizes at the sites of DNA damage and its silencing impairs the DDR

**Figure 2.**
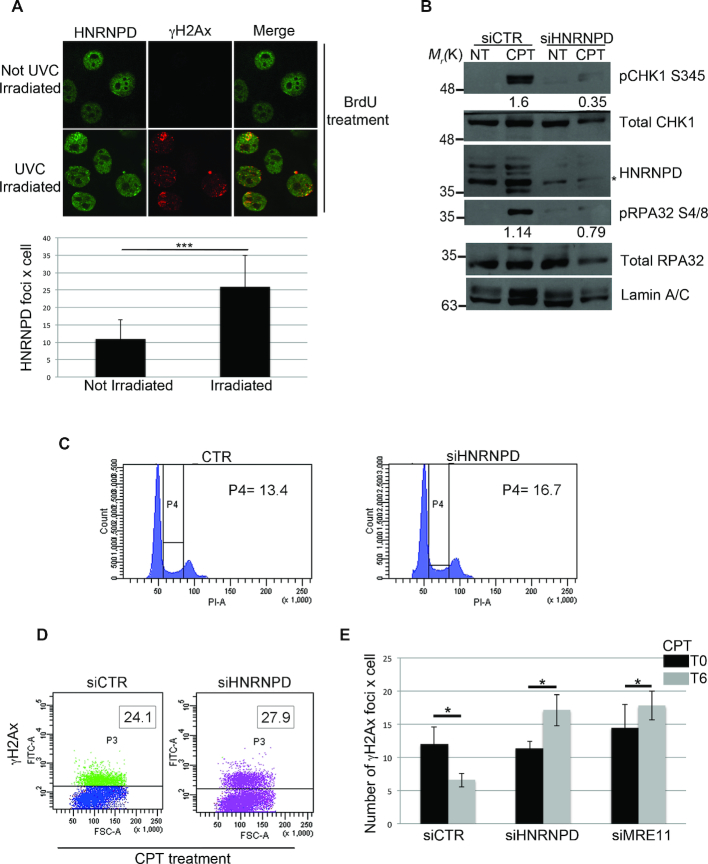
HNRNPD regulates the DNA damage response. (**A**) HeLa cells were incubated with 10 μM BrdU for 16 h followed by exposure to 50 J/m^2^ of UVC to induce localized DSBs, and cells were allowed to incubate for 1 h before fixation. Immunofluorescence was performed with the indicated antibodies. Lower panel: ImageJ analysis of laser irradiation images to quantify the number of HNRNPD foci × cell before and after irradiation. Data represent the mean ± standard deviation (s.d.). (*n* = 3 independent experiments). *P-value <0.05. (**B**) Western blot analysis with the indicated antibodies of HeLa cells, 48 h after transfection with siHNRNPD or siCTR followed by 2-h treatment with 1 μM CPT. GAPDH was assessed as a normalization control. The values of band density corresponding to pCHK1 S345 and pRPA32 S4/8, normalized to the total protein levels, are reported. (**C**) Cell cycle profile of HeLa cells, 48 h after transfection with siHNRNPD or siCTR, was analyzed through flow cytometry upon propidium iodide (PI) staining. (**D**) γH2Ax values were measured by FACS analysis in HeLa cells, transfected with the indicated siRNAs for 48 h, then treated with 1 μM CPT for 2 h. The insets report the percentage of γH2Ax positive cells. (**E**) HeLa cells were transfected with the indicated siRNAs for 48 h, treated with 1μM CPT for 2 h (T0) followed by drug washout and incubation in CPT-free medium for additional 6 h (T6). γH2AX foci were measured at T0 and T6 with the ImageJ software analysis. Data represent the mean ± s.d. (*n* = 4 independent experiments). *P-value <0.05.

The ability of DNA repair factors to re-localize to DNA damage sites is a prerogative for the correct DDR. So, to test whether HNRNPD can move from undamaged DNA to specific damage sites, we used micro irradiation coupled with BrdU treatment, to induce localized DSBs (23). Interestingly, 30 min after 25 J/m^2^ of UVC laser irradiation, we observed an increased number of HNRNPD foci most of which showed colocalization with γH2Ax (Ser139) foci (Figure 2A). The DDR is regulated by multiple phosphorylation events, which include signals for the repair factors ultimately involved in the processing of the DNA lesions. The down-regulation of any of these proteins can affect genome stability (47). Here, to gain insight into the function of HNRNPD within the DDR, we silenced HNRNPD expression in HeLa cells using specific siRNAs and analyzed the expression of key DDR players in response to CPT. We found that, upon CPT treatment and with respect to control cells (siCTR), HNRNPD silenced cells (siHNRNPD) had reduced levels of CHK1 S345 phosphorylation, a crucial event for the activation of the DNA damage checkpoint, suggesting that HNRNPD silencing impairs checkpoint activation (Figure 2B). Moreover, the phosphorylation of RPA32 S4/8, a marker of replication checkpoint activation and, importantly, of DNA end-resection (11,48,49), was reduced in HNRNPD silenced cells, as shown by western blot analysis (Figure 2B). Importantly, HNRNPD silenced cells showed a similar cell cycle profile compared with control cells, indicating that the down-regulation of RPA32 phosphorylation, in siHNRNPD cells, could not be reasonably explained by cell cycle-dependent differences as opposed to HNRNPD knockdown (Figure 2C). The CPT treatment induces activation of γH2Ax and MRN complex for the proper repair of the DNA DSBs (50). We analyzed the impact of HNRNPD silencing on the DNA repair kinetics, measured as γH2Ax foci resolution upon the DNA damage induction. First, we showed a normal γH2Ax activation in response to DNA damage, upon 30 min of CPT in siHNRNPD cells compared to siCTR, consistent with correct lesion recognition (51) (Figure 2D). Moreover, upon 6 h from CPT washout, HeLa siCTR cells reduced the γH2Ax foci up to 50%. Conversely, HeLa HNRNPD silenced cells showed an impaired foci resolution, as for the siMRE11 cells, consistent with inability to repair the DSBs (Figure 2E). We have not assessed here the effect of HNRNPD silencing on γH2Ax foci formation in basal conditions (no DNA damage induction) although a possible role in telomere dysfunction-induced foci has been previously found (52).

Overall, these data further support a possible involvement of HNRNPD in the DNA damage response and in the processing of the DNA lesions.

### HNRNPD is involved in the DNA end-resection process

**Figure 3.**
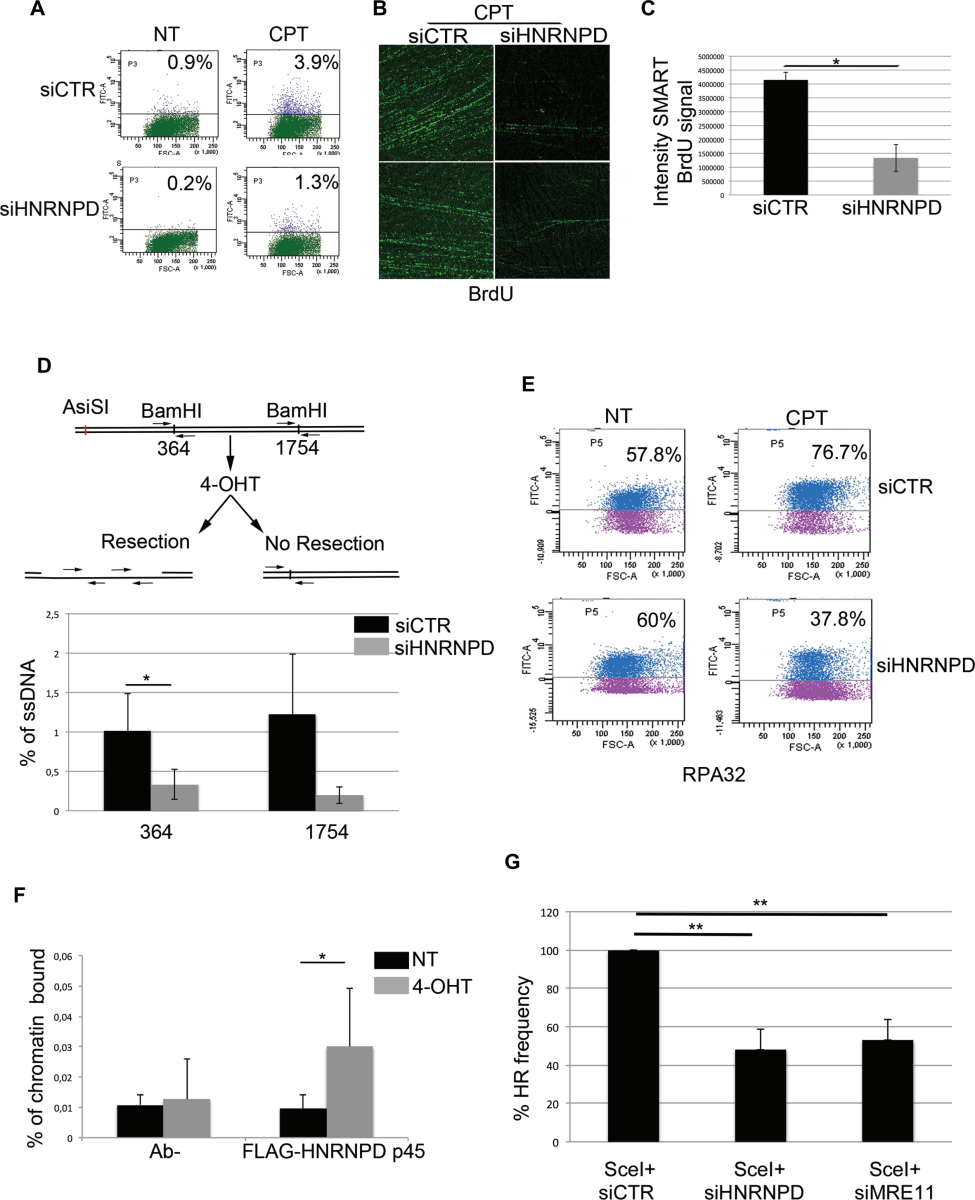
HNRNPD regulates the DNA end-resection process. (**A**) FACS analysis of ssDNA formation in HeLa cells transfected with siCTR or siHNRNPD for 24 h, then pulse-labelled with 10 μM BrdU for additional 24 h and treated with 1 μM CPT for 2 h. HeLa cells were prepared for cytofluorimetric analysis in non-denaturing conditions in order to quantify the percentage of positive ssDNA cells. The untreated condition (no DNA damage) reveals the background level given in each cell type by the FITC-antibody detecting BrdU (in this case BrdU is entrapped within the double helix and cannot be recognized by the antibody). Such background values, subtracted to the values obtained following CPT treatment, indicate a ∼3-fold decrease in single strand DNA upon HNRNPD silencing. (**B**) To perform the SMART technique, HeLa cells (both siCTR and siHNRNPD) were pulse-labelled with 10 μM BrdU for 24 h then treated with 1 μM CPT for 2 h before spreading onto Silane Prep Slides. Immunofluorescence was performed with the BrdU antibody. Two representative images for each cell type (siCTR and siHNRNPD) are shown. (**C**) BrdU signal intensity of SMART technique analyzed by ImageJ software. Data represent the mean ± standard deviation (s.d.). (*n* = 2 independent experiments). *P-value <0.05. (**D**) HeLa ER-AsiSI were transfected with siCTR or siHNRNPD for 48 h followed by treatment with 300 nM of 4-OHT. The Real-time qPCR was performed on the genomic DNA with the indicated primers amplifying regions including the positions at 364 and 1754 bp downstream from the DSB, as depicted. The values of %ssDNA are reported as the mean ± s.d. (n = 4 independent experiments) and were calculated as follows: %ssDNA = 1/[(2^ΔCt^-1) + 0.5] × 100 (83). **P-value <0.01; *P-value <0.05. (**E**) HeLa cell lines were transfected with the indicated siRNAs for 48 h treated or not with 1 μM CPT for 2 h; before fixative, cells were pre-extracted with the CSK + 0.1% Triton X100 + 0.3 μg/ml RNase A followed by incubation with indicated antibody. RPA32 values were analyzed through flow cytometry. (**F**) ChIP experiments of HeLa ER-AsiSI, treated or mock treated with 300 nM 4-OHT for 1 h, were performed by using either an HNRNPD antibody or control IgGs (Ab-). The HNRNPD chromatin binding ability was measured, as a percentage of immunoprecipitated input, from qPCR values of an 80 bp amplicon including the AsiSI site. Data represent the mean ± s.d. (*n* = 4 independent experiments). *P-value <0.05. (**G**) HeLa DR-GFP cells were transfected with the plasmid encoding the SceI restriction enzyme in presence of siCTR, siHNRNPD or siMRE11 followed by FACS analysis measurement of GFP levels used to calculate %HR frequency compared with siCTR which was set as 100%. Data represent the mean ± s.d. (*n* = 3 independent experiments), **P-value <0.01.

The HR repair pathway requires DNA end-resection in order to produce ssDNA tails needed for the invasion of the complementary strand used as template for an error-free repair (53). To assess HNRNPD role in this process, we first analyzed, by cytofluorimetric analysis, the BrdU accumulation under non-denaturing condition as a surrogate marker of ssDNA formation upon CPT (9). We found a reduction of ssDNA formation in HNRNPD silenced HeLa cells, compared with control cells, following 48 h of silencing and 24 h of BrdU labelling, upon 1 h of drug treatment (Figure 3A and Supplementary Figure 2a). HNRNPD protein depletion seemed to impair ssDNA formation only in the DNA damage condition and not following replication stress (Supplementary Figure 2b). We used the SMART technique (27) to gain a snapshot visual assessment of the effect of HNRNPD silencing on ssDNA upon CPT. Indeed, HNRNPD silencing markedly reduced BrdU bulk immunofluorescence upon CPT treatment compared with the siCTR cells (Figure 3B and C). So, we asked whether HNRNPD down regulation affects the DNA end resection process (54) and decided to assess more precisely the extent of ssDNA formation; to this aim, we generated HeLa cells expressing the inducible ER-AsiSI system to create DSBs *in vivo* at specific loci (28). Cell treatment with 4-hydroxytamoxifen (4-OHT) induces the nuclear localization of the engineered AsiSI endonuclease followed by DSBs, repaired through the DNA end-resection and HR (28). The percentage of resected DSBs was calculated by qPCR using two sets of primers, amplifying two regions that include BamHI sites located at 364 and 1754 bp from the AsiSI break site and therefore able to identify a quite wide range of resection. Surprisingly, HNRNPD knockdown markedly reduced the efficiency of DNA end-resection at both sites, positioning HNRNPD upstream to this process (Figure 3D and Supplementary Figure 2c). As a control, given that HNRNPD is one of the principal RNA binding protein involved in mRNA fate (40), we checked whether its silencing could affect the mRNA and protein levels of key players underlying the DNA end resection process. We found that HeLa siHNRNPD cells showed unperturbed levels of MRE11, EXO1, CTIP, (Supplementary Figures 2d–f).

The extended ssDNA tails, produced through DNA end-resection, are rapidly coated by the heterotrimeric RPA complex. Consistent with the affected DNA end-resection process, HeLa HNRNPD silenced cells showed an impaired RPA32 DNA binding compared with the control cells in response to CPT, measured by FACS analysis (Figure 3E) (24). Indeed, HNRNPD silencing in HeLa ER-AsiSI reduced RPA32 DNA binding ability upon DNA damage induction measured through ChIP analysis (Supplementary Figure 2g). Similarly, upon transfection of the FLAG tagged HNRNPD p45 protein isoform and DSB induction, we demonstrated HNRNPD increased presence near the region proximal to the AsiSI cut sites through ChIP analysis (Figure 3F and Supplementary Figure 2h). Finally, we generated HeLa cell lines stably expressing the pDR-GFP system in order to verify the impact of HNRNPD down-regulation on HR efficiency (19,20). The transfection of the I-SceI endonuclease in pDR-GFP cells induces DSBs, which activate the endogenous HR repair pathway that in turn leads to functional GFP reconstitution; the co-transfection with siRNAs against HNRNPD reduced the HR efficiency of ∼50% compared with the control siRNAs and similarly to the effect achieved with MRE11 downregulation (Figure 3G and Supplementary Figure 3a). Collectively, these data suggest a possible role of HNRNPD in the regulation of DNA end-resection, probably in the initial steps of the process.

### HNRNPD knockout sensitize HeLa cells to PARP inhibition

**Figure 4.**
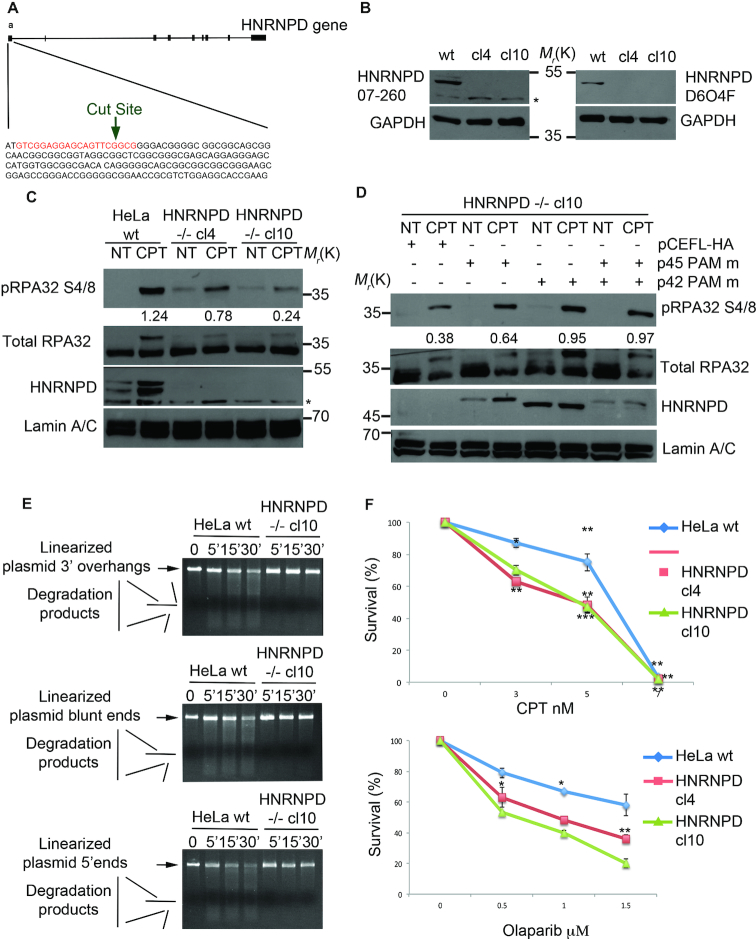
HNRNPD knockout affects the DNA damage response and sensitizes cells to CPT and olaparib treatment. (**A**) Schematic representation of the CRISPR/Cas9 strategy used to develop the HNRNPD ko cells. The sgRNA guide is indicated in red. (**B**) Western Blot analysis of HeLa HNRNPD ko clones with either the Millipore (07-260, left panel), or the Cell signalling (D6O4F, right panel), antibody. The asterisk indicates a non-specific band, which was detected by the Millipore antibody. GAPDH was used as a loading control. (**C**) Western blot analysis of DNA damage response markers following treatment with 1 μM CPT for 2 h in HeLa wt cells and HNRNPD knockout cell clones. Lamin A/C was used as a loading control. The values of band density corresponding to pRPA32 S4/8, normalized to the total protein levels, are reported. (**D**) HeLa HNRNPD–/– cl10 cells were transfected with the pCEFL-HA HNRNPD isoforms with the PAM mutated sequence for 48 h followed by 1μM CPT for additional 2 h followed by western blot analysis with the indicated antibodies. Different expression levels are likely dependent on either a different transfection efficiency or by plasmids co-transfection. Lamin A/C was used as a loading control. The values of band density corresponding to pRPA32 S4/8, normalized to the total protein levels, are reported. (**E**) DNA end-resection assay *in vitro* with the nuclear protein extracts from HeLa wt and HNRNPD cl10 cell lines. Linearized plasmid with either 3′, blunt or 5′ overhangs were incubated with protein extracts for the indicated time points and run on a 0.8% agarose gel + EtBr. (**F**) HeLa and HNRNPD cell clones were treated with the indicated drugs at crescent concentrations for ten days followed by staining with crystal violet. Asterisks indicate: ***P-value <0.001; **P-value <0.01; *P-value <0.05, respectively.

To verify and confirm the above experiments, we generated, through the CRISPR/Cas9 system, HNRNPD–/– HeLa cell clones (Figure 4A). Western blot analysis showed the knockout of all HNRNPD protein isoforms in two HeLa HNRNPD–/– clones (Figure 4B). In order to confirm the alteration of DNA end-resection markers, as observed in HeLa siHNRNPD cells (Figure 2b), we treated HNRNPD–/– cells with CPT followed by western blot analysis showing a marked reduction of both RPA32 S4/S8 and CHK1 S345 phosphorylation (Figure 4C and Supplementary Figure 3b, respectively). To assess whether HNRNPD re-expression in HeLa HNRNPD–/– cell clones recovers the DDR function, we generated pFLAG-HNRNPD expression vectors with the four isoforms mutated in the PAM sequence (to avoid Cas9 cut). The expression of different HNRNPD isoforms (p45, p42, p40) proved able to increase pRPA32 S4/8 phosphorylation, upon CPT treatment and each functioned in combination with p45 (Figure 4D and Supplementary Figure 3c), whereas p37 (Supplementary Figure 3d) did not. To demonstrate the direct involvement of HNRNPD in the DNA resection process, we performed an *in vitro* assay using digested plasmids with either 3′ protruding, 5′ protruding or blunt ends and incubated them with the nuclear extract derived from wt HeLa or HNRNPD–/– cells. Remarkably, HNRNPD loss abolished the DNA degradation ability observed with the wt extracts (Figure 4E, Supplementary Figure 3e–g).

Finally, to assess the potential clinical relevance of HNRNPD, we assessed whether its knockout could sensitize HeLa cells to treatment with CPT, the precursor of a broad class of antineoplastic drugs widely used for the treatment of several tumours (55). So, we performed a clonogenic assay and found that both the HNRNPD–/– clones cl4 and cl10 showed a slightly increased sensitivity to the cytotoxic effects of CPT resulting in a 6 nM IC50 for wt HeLa compared with 4.5 nM IC50 for both cl4 and cl10 HNRNPD–/– clones (Figure 4F). As HR proteins, such as BRCA1/2, have been described to be synthetic lethal with the inhibition of other repair factors, such as the poly (ADP-Ribose) polymerase inhibitor 1, PARP1 (56), we then treated both wt HeLa cells and the two HNRNPD–/– clones, with the PARP1 inhibitor olaparib. Indeed, we found that the absence of HNRNPD sensitized HeLa cells to olaparib-induced growth inhibition. Interestingly, olaparib, at the highest concentration tested, remarkably reduced clonogenic ability in HeLa cells null for HNRNPD whereas it did not reach 50% inhibition of colony formation in wt HeLa cells (Figure 4F). Taken together, these data suggest that HNRNPD modulation could be used to sensitize cancer cells to various antitumoral agents. It will have to be further established whether re-expression of HNRNPD (or specific isoform combinations) in knockout cells is able to recover the DNA repair ability (in DNA end resection assays) and reverse CPT and olaparib sensitivity.

### HNRNPD interacts with SAF-A and its silencing induces R-loop accumulation nearby the DNA lesions

**Figure 5.**
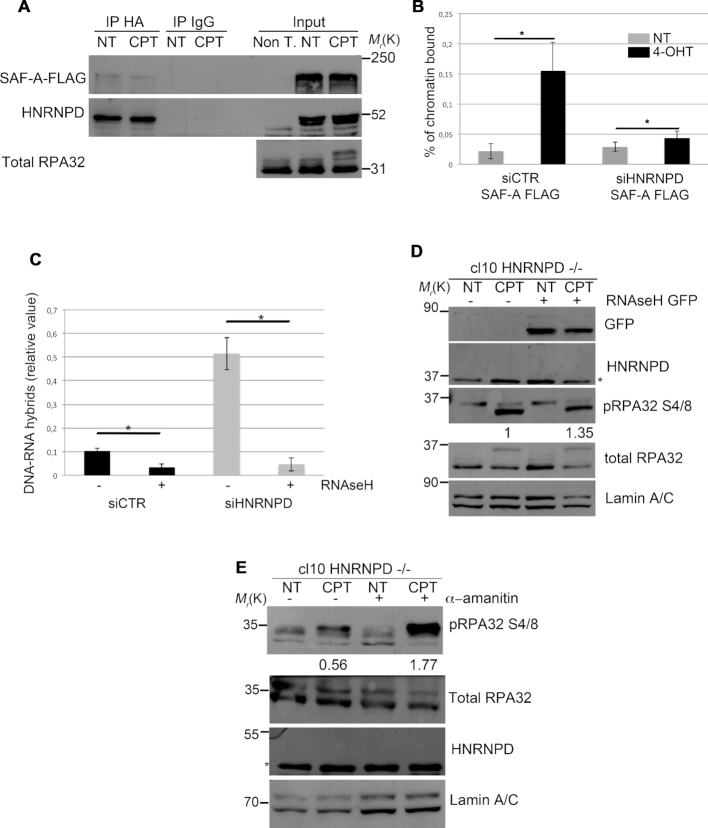
HNRNPD regulates R-loops through the localization of SAF-A protein. (**A**) HeLa cells were transfected with either SAF-A FLAG or HNRNPD-HA, incubated for 48 h and treated or not for 2 h with 1μM CPT. Protein lysates were incubated 30 min on ice with 0,3 μg/ml of RNase A and 50 μg/ml of EtBr followed by centrifugation to remove the debris. Immunoprecipitation was conducted with anti-HA antibody over night at +4°C. An antibody against total RPA32 was used as a DNA damage control. (**B**) HeLa ER-AsiSI were transfected with the indicated siRNAs in combination with the plasmid coding for SAF-A-FLAG for 48 h and treated or mock treated with 300 nM 4-OHT for 1 h. ChIP analysis was performed by using either an anti FLAG antibody or control IgGs (Ab–). SAF-A chromatin binding ability was measured as a percentage of immunoprecipitated input, from qPCR values of a 200 bp amplicon upstream to AsiSI cut site. Data represent the mean ± s.d. (*n* = 3 independent experiments). *P-value <0.05. (**C**) HeLa cells were transfected with the indicated siRNAs for 48 h followed by induction of DSBs through the AsiSI enzyme. The immunoprecipitation was carried out with the anti-DNA:RNA hybrid S9.6 antibody over night at +4°C. Data represent the mean ± s.d. (*n* = 3 independent experiments). *P-value <0.05. (**D**) HeLa HNRNPD ko cell line cl10 was transfected with RNaseH GFP coding plasmid and incubated 48 h followed by 1 μM CPT treatment and western blot analysis. Lamin A/C was used as a normalization control. Densitometric analysis of pRPA32 S4/8 activation was carried out through the ImageJ software. (**E**) HeLa HNRNPD ko cl10 was treated with 10 μM of α-amanitin for 6 h followed by co-treatment with 1 μM CPT (where indicated) for additional 2 h followed by western blot analysis. Lamin A/C was used as a normalization control. Densitometric analysis of pRPA32 S4/8 activation was carried out through the ImageJ software.

The heterogeneous nuclear ribonucleoproteins regulate various aspects of cell biology such as mRNA export, localization, DNA repair and resolution of R-loop structures (57–59). One of these, previously involved in the DNA damage response, is HNRNPU (also known as SAF-A) (59). So, we investigated whether SAF-A and HNRNPD could possibly interact. We transfected HeLa cells with vectors expressing SAF-A-FLAG and HNRNPD-HA and found through reciprocal co-immunoprecipitation experiments that the two proteins are indeed able to interact (Figure 5A and Supplementary Figure 4a). The protein lysates, before the immunoprecipitation, were treated with RNase A and EtBr to remove mRNA and DNA, respectively. Through ChIP assay, near the AsiSI cut sites, we found reduced SAF-A localization upon DSB induction in HNRNPD silenced cells compared with control cells (Figure 5B). Consistently, by means of chromatin enriched purification, in the presence of RNase A, we confirmed a reduced chromatin loading of SAF-A upon DNA damage in HNRNPD knockout cells (Supplementary Figure 4b), suggesting that HNRNPD silencing affects SAF-A localization on the damaged DNA.

As SAF-A dynamics (presence/exclusion) on the damaged sites was associated with R-loop modulation (59), we performed a DRIP assay to analyze R-loops near the DSB induced by AsiSI in HNRNPD silenced cells (Figure 5C). Quantitative RT-PCR analysis (with a primer pair amplifying a DNA region distant ∼600 bp from the enzyme cut site) showed an increased number of R-loops in siHNRNPD cells compared with the control cells.

Recently, R-loops were described to be essential regulator of DNA end-resection favouring the DDR, although with a seemingly contrasting function (60,61). Indeed, while DNA:RNA hybrid presence on damaged DNA seems to promote repair (likely in an earlier phase), their proper removal is also required to this aim. In fact, overexpression of RNase H1, which digests the RNA within DNA:RNA hybrids thereby reducing R-loop occurrence, reduced DNA resection and repair (60). So, we analyzed the effect of RNase H1 ectopic overexpression in HNRNPD KO cells upon CPT treatment (Figure 5D). RNase H1 expression was able to recover RPA32 S4/8 phosphorylation defects, further supporting the idea that HNRNPD acts favouring R-loop removal for a proper DNA-end resection. Moreover, as the RNA pol II generates the mRNA that can invade the duplex of resected DNA favoring R-loop formation (61), we assessed whether the selective inhibition of RNA pol II through α-amanitin could also rescue the defective RPA32 S4/8 phosphorylation upon DNA damage observed in HNRNPD KO cells. Indeed, α-amanitin pre-treatment (62) functioned similarly to RNase H1 in rescuing RPA32 S4/8 phosphorylation upon damage in cells lacking HNRNPD function (Figure 5E). Overall, these data argue for a crucial role of HNRNPD in the R-loop resolution, possibly contributing at least to some extent with SAF-A recruitment, for a proper DDR.

## DISCUSSION

DNA damage repair defects are a leading cause of genomic instability, which is one of the main features enabling cancer development (63). Many sources of DNA damage, endogenous or environmental, can harm the genome activating the cellular response. DNA repair defects predispose to various diseases such as cancer and accelerate aging (64). Recently, RBPs were described to regulate the DDR both through their functions within RNA biology (such as splicing regulation) (65) and in the processing of DNA lesions (66). Among these, the RBP HNRNPD was shown to regulate the mRNA fate of many DDR proteins in response to DNA damage (40). The alteration of HNRNPD expression was found to affect many genes underlying tumorigenic phenotypes owing to deregulation of their post-transcriptional control (67); interestingly, HNRNPD knockout mice showed accelerated aging associated with telomere erosion, increased inflammatory cytokines and cellular senescence (52).

Here, we performed a proteomic screening, using a synthetic DNA end-resection intermediate, and identified HNRNPD as a new player, directly involved, in the DDR. HNRNPD has four different splicing isoforms (p45, p42, p40, p37) and we identified, through LC-MS analysis, peptides corresponding specifically to the p45 and p40 proteins. However we found that all HNRNPD isoforms are able to bind the HeLa chromatin and the synthetic biotinylated DNA. Consistently with our data HNRNPD was recently identified, along with the MRN complex, as bound to damaged DNA in *Xenopus l*. egg extracts (68). Indeed, HNRNPD proved able to bind the synthetic structure *in vitro* and the binding was independent of the RPA complex; the HNRNPD binding was not sequence specific but dependent on free ends, consistent with a possible recognition of randomly induced DNA DSBs. The reduced binding of HNRNPD to the biotin-tagged ends demonstrated the reliability of the screening methods and the specific binding of the protein to the DNA. We also found that HNRNPD was able to bind chromatin DNA, which is a prerogative of DNA repair factors (69). By generation of HNRNPD deletion mutants we demonstrated that its DNA binding activity, both *in vivo* and *in vitro*, was not dependent on RRMs, which are responsible of the HNRNPD binding to the human single strand telomeric repeats (45). Here, we found that the DNA binding ability of HNRNPD required the C-terminal protein domain. We have not been able to achieve expression of a mutant containing the C-terminal only, devoid of RRM motifs, to demonstrate formally the DNA binding ability of the sole C terminal domain. However, the use of RNase A in all the DNA binding experiments suggests that HNRNPD binds DNA independently of its (RRM-mediated) RNA regulatory function. Indeed, HNRNPD was previously classified as a DNA- and RNA-binding protein (DRBP) (70). Interestingly, the C-terminal domain contains a glycine–arginine rich domain (GAR) (46); this domain is common to many proteins with different functions including MRE11, in which the methylation of the arginine within the GAR motif is essential for its localization to the DSBs *in vivo* (71). Intriguingly, the regulation of GAR methylation depends on the family of protein arginine methyltransferases (PRMTs) of which PRMT6 is responsible for HNRNPD protein methylation (72). However, DNA damage did not increase HNRNPD chromatin fraction suggesting that, upon damage, HNRNPD re-localizes from unperturbed DNA to damage sites. Consistently, HNRNPD was able to re-localize on the DNA damaged induced foci suggesting a possible role in the recognition of the lesion favouring its repair. This would be in accordance with the correct γH2Ax activation and the impaired foci resolution observed in siHNRNPD HeLa cells, in a manner similar to what occurs in siMRE11 HeLa cells. In this scenario, it seems possible that post-translational modifications of HNRNPD, within or not the C-terminal domain, might direct its re-localization onto DNA damage sites and affect the DNA binding activity.

The DNA end-resection process is characterized by protein localization, on the sites of damage, which are essential for the generation of the ssDNA tail rapidly coated by RPA complex in order to promote the RAD51 loading and the strand exchange (73). Although we did not investigate the temporal loading of HNRNPD with regard to the DNA end-resection cascade, we demonstrated its direct involvement in the process showing impairment of ssDNA formation upon CPT treatment, assessed through BrdU analysis, but not upon the replication stress induced by the HU treatment. Indeed, FACS analysis showed that HNRNPD silencing in HeLa reduced, upon DNA damage, the chromatin localization of RPA32, readout of ssDNA formation. In particular, HNRNPD silencing affected DNA-end resection at both 364 and 1754 nucleotides from the AsiSI-induced DSBs, without altering the mRNA levels of the principal players underlying this process. The ChIP analysis of HNRNPD onto the DSBs sites induced by the AsiSI enzyme, confirmed the physical presence of the protein near the DNA lesions. Consistently, HNRNPD silencing reduced the cell ability to repair the DSBs through the HR, measured by a GFP reporter assay (20), mirroring the effect of MRE11 silencing (74). The reduced phosphorylation of CHK1 and RPA32, in HNRNPD silenced cells suggest its involvement in the processing of the lesions rather than in its signalling as confirmed by the normal activation of the DNA damage signalling marker γH2Ax (51). Moreover, the unaffected cell cycle distribution of HeLa siHNRNPD with respect to siCTR cells confirms that the checkpoint impairment was not due to an indirect effect.

Consistently with HNRNPD silencing, the CRISPR-Cas9 mediated HNRNPD knockout also affected the DDR by reducing CHK1 S345 phosphorylation and, in particular, the end-resection process as shown by reduced RPA32 S4/8 phosphorylation and impaired DNA resection *in vitro*. HNRNPD silencing affected ssDNA formation upon DNA damage induced by CPT but not upon DNA replication block through HU, suggesting a specific role of HNRNPD in DNA repair. Interestingly, HNRNPD knockout increased cell sensitivity to both CPT and the PARP1 inhibitor olaparib, as occurs with loss of other proteins involved in HR such as BRCA1/2–/– cells (75), providing the framework for a possible use of HNRNPD modulation to sensitize cancer cells to widely used antitumoral agents. Various mechanisms have been proposed to explain why HR defective cells (such as BRCA1/2–/–) develop sensitivity to inhibitors of PARP1 activity beyond the original model suggesting that PARP1 inhibition increases the rate of single strand breaks, which, during replication, are converted to DSBs thus implying repair through HR (76). Despite the underlying mechanism involved, the fact that HNRNPD knockout sensitized cells to PARP1 inhibition through olaparib strongly supports its role in HR herein uncovered (76).

The HNRNPs family represents a large protein family involved in many cellular processes and diseases, such as neurodegenerative diseases and cancer (77,78). These proteins act within mega- or mini-complexes comprising two or more HNRNPs, providing them a functional versatility. The best characterized role of HNRNPs family is the regulation of mRNA processing (57); recently, additional functions were described including a role in the DNA damage response (58,59). In particular, HNRNPUL1 was described to be involved in the regulation of DNA end-resection downstream of MRE11 and CTIP favouring the loading of BLM protein. More recently, BLM-DNA2 mediated long-range resection was found to be regulated by CTIP (79). Here, we found that the localization of HNRNPD to the damaged sites was independent of MRE11, CTIP or EXOI because it was not affected by their silencing (data not shown). So, our findings seem to suggest a role of HNRNPD upstream the DNA-end resection process, independently of MRE11, CTIP and EXOI. However it will have to be determined whether HNRNPD impacts on MRN complex recruitment onto damaged sites.

Emerging evidences have implicated R-loop structures in the DNA damage response (15,80) and RNA binding proteins in the regulation of these structures (59). SAF-A, another HNRNP, was previously involved in the regulation of DNA repair and associated with the regulation of R-loop structures (59). Here we found that HNRNPD and SAF-A are able to physically interact in a way that seems independent of DNA and/or mRNA presence. The RBD domain of SAF-A was previously found important for the exclusion of the HNRNP from damaged sites and the authors performed LC-MS analysis of SAF-A-RBD to identify putative protein partners (59). HNRNPD was not found in this screening probably because the small SAF-A-RBD used as bait is not necessary for the interaction. Indeed, our data indicate that SAF-A/HNRNPD interaction requires the SPRY domain of SAF-A (data not shown), although interaction between HNRNPD and SAF-A endogenous proteins still needs to be assessed. Interestingly, SAF-A localizes onto DNA breaks with a peculiar dynamics and correlates with DNA:RNA hybrid resolution (59). We herein demonstrated an increased amount of DNA:RNA hybrids in siHNRNPD HeLa cells with respect to the controls at the AsiSI induced DSBs sites, measured through the DRIP analysis. We also observed a reduced chromatin DNA loading of SAF-A protein in response to CPT treatment in the absence of HNRNPD. Consistently, SAF-A ability to localize near the AsiSI-induced DSBs was reduced in HeLa siHNRNPD cells, measured through ChIP assay. Overall these data further support a role of heterogeneous nuclear ribonucleoproteins in the regulation of R loops at damaged sites, which deserves further investigation.

**Figure 6.**
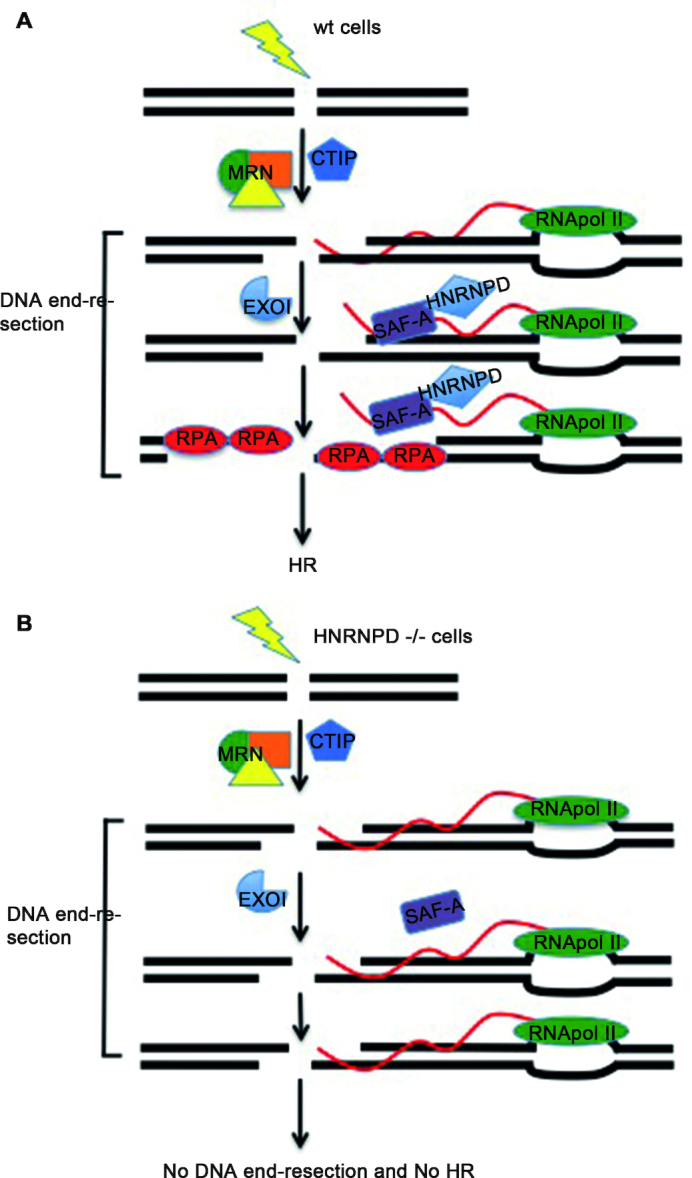
Schematic representation of the hypothesized HNRNPD mechanism of action during homologous recombination. (**A**) In HeLa wild type cells, upon DNA damage, HNRNPD is relocated to DNA damage sites. Upon damage also SAF-A is located near the DNA lesion and both proteins are associated with a proper removal of the R-loop structures during the DNA end-resection process. (**B**) In HNRNPD silenced cells, SAF-A does not localize onto damaged sites and R-loops accumulate impairing the DNA end-resection and ultimately the homologous recombination process.

The relevance of DNA:RNA hybrid resolution for a proper DNA repair has been recently described. It has been shown that the formation of R-loop at the DSBs is facilitated by the initial 5′-end resection, operated by the MRN complex, and, consistently, the inhibition of RNase H activity in *S. Pombe* decreased RPA loading, near the DSBs (81). The crosstalk between RNase H and RPA is thought to be evolutionary conserved (33). In light of these findings the reduced RPA32 loading, measured by FACS and ChIP analysis upon DNA damage, which we observed in siHNRNPD cells, is likely indicative of a defective R-loop resolution. Indeed, ectopic expression of RNase H1 or α-amanitin treatment restored RPA32 S4/8 phosphorylation in HNRNPD KO cells suggesting that R-loop accumulation impairs HNRNPD-mediated DNA end resection. The underlying mechanism whereby HNRNPD regulates R loop structures, upon DNA damage but possibly also in the basic transcriptional setting, however, still needs to be characterized. Interestingly a recent study found HNRNPD (as well SAF-A) as part of the DNA:RNA hybrid-interactome identified through mass spectrometry analysis (82) further supporting their possible role in R loop regulation. In summary, we identified HNRNPD as a novel factor involved in the DNA end-resection process, a key step essential for the HR-mediated repair, associated with proper R-loop resolution (Figure 6).

## Supplementary Material

Supplementary Data
