# Supplementary material for: Depletion of the RNA binding protein HNRNPD impairs homologous recombination by inhibiting DNA-end resection and inducing R-loop accumulation
Source: Nucleic Acids Res. 2019 Feb 25;47(8):4068–85. doi: 10.1093/nar/gkz076 (PMC6486545; doi:10.1093/nar/gkz076)
Supplement: Supplementary Data [file gkz076_supplemental_files.zip › Supplementary Data.pdf]

a

PROTEIN NAME	AC	Protein	Mascot search results	
			**Identified Peptide Sequence**	**Score**
**DNA ds RPA WT**				
Heterogeneous nuclear ribonucleoprotein D0	Q14103	HNRPD_HUMAN	1) FGEVVDCTLKLDPITGR<br>2) MFIGGLSWDTTKK<br>3) HSEAATAQREEWK	62<br>33<br>34
Heterogeneous nuclear ribonucleoprotein A1	P09651	ROA1_HUMAN	NQGGYGGSSSSSSYGSGR	100
X-ray repair cross-complementing protein 6	P12956	XRCC6_HUMAN	IMATPEQVGK<br>TFNTSTGGLLLPSDTKR<br>IMLFTNEDNPHGNDSAK<br>NIYVLQELDNPGAK<br>SDSFENPVLQQHFR	49<br>53<br>34<br>69<br>42
Replication protein A 70 kDa DNA-binding subunit	P27694	RFA1_HUMAN	SGGVGGSNTNWK<br>ENCMYQACPTQDCNKK	39<br>36
Replication protein A 32 kDa subunit	P15927	RFA2_HUMAN	IGNVEISQVTIVGIIR	52
Replication protein A 14 kDa subunit	P35244	RFA3_HUMAN	IIHDFPQFYPLGIVQHD	35

The table lists all the peptides/proteins, identified through LC/MS having significant Mascot scores with the parameters indicated in the “Materials and Methods” section. Such low number of proteins, which includes however known players of the DDR, might be due to the low sensitivity of the instrument.

b

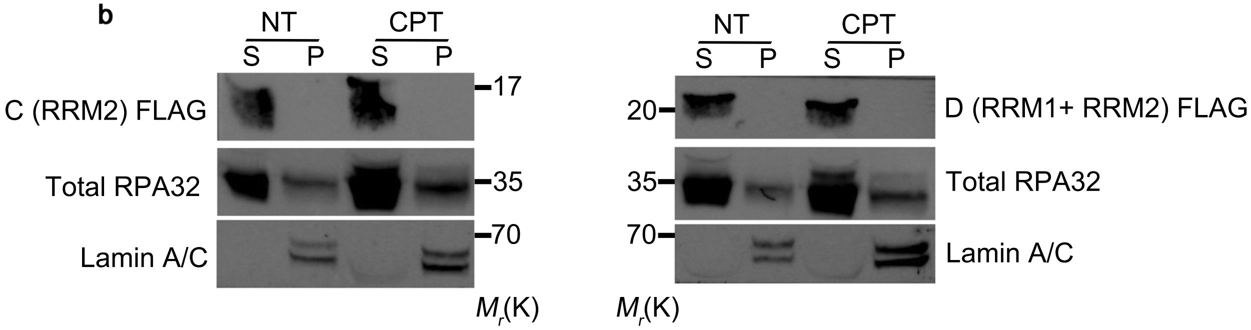

c

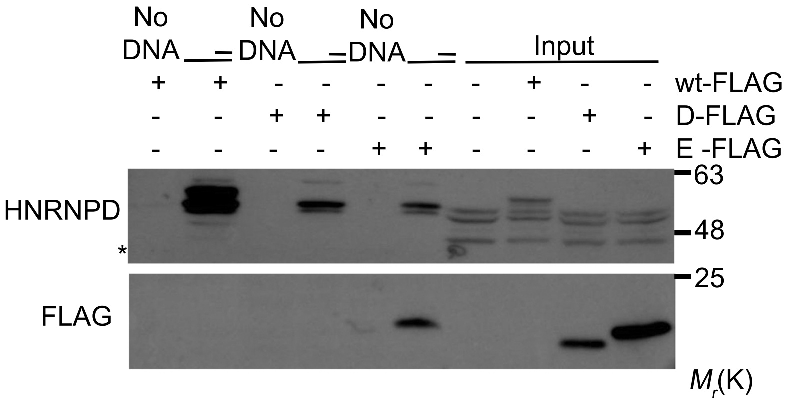

d

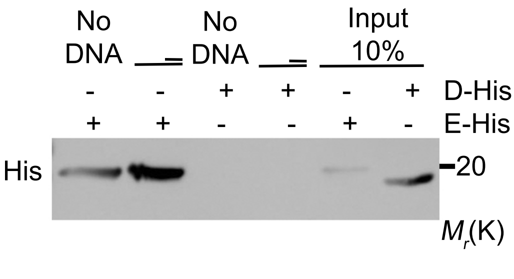

Supplementary Figure S1

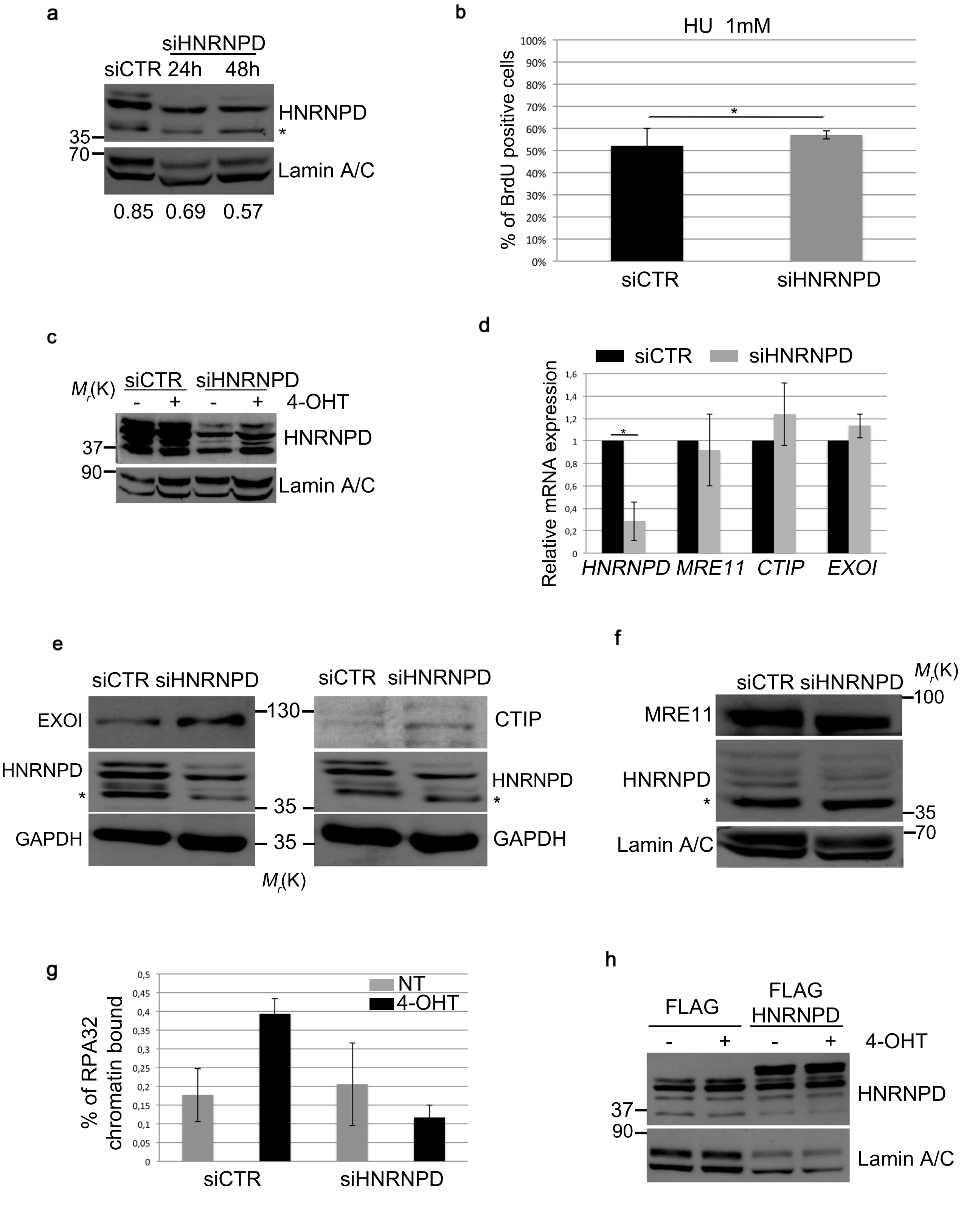

Supplementary Figure S2

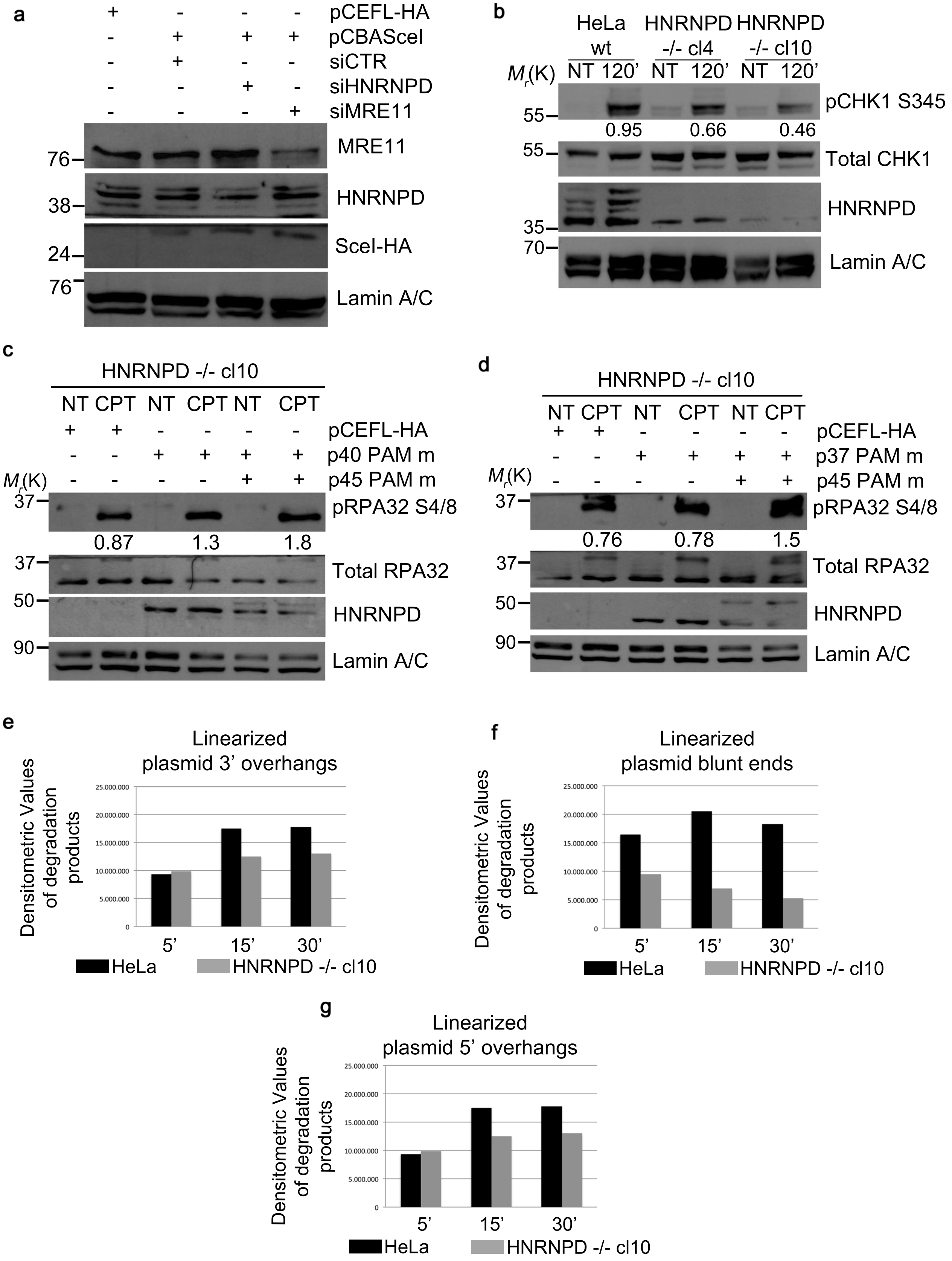

Supplementary Figure S3

a

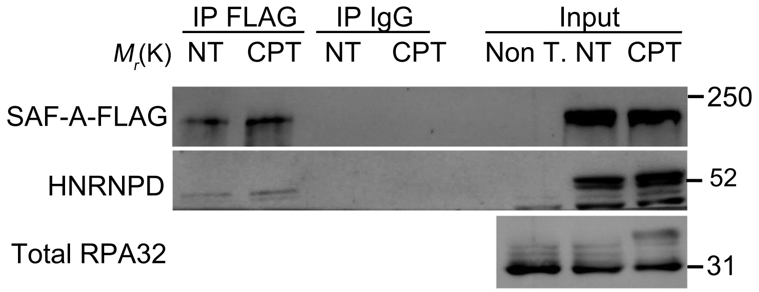

b

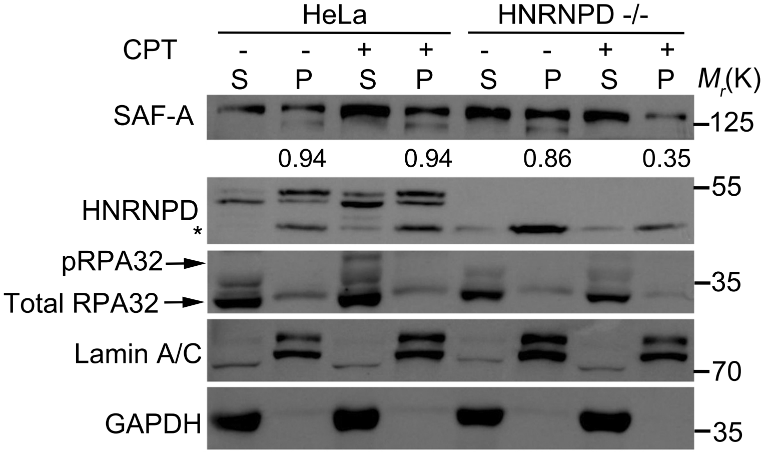

Supplementary Figure S4

Supplementary Figure 1. Assessment of HNRNPD binding to synthetic DNA structures and chromatin.

(a) List of peptides obtained from the proteomic screen along with their corresponding proteins. **(b)** Chromatin enriched purification of HeLa cells transfected with p45-RRM2-FLAG and p45-RRM1+RRM2-FLAG encoding plasmids treated or not with 1µM CPT. Here we loaded 120 µg of protein extract for each line. Lamin A/C was used as a chromatin enrichment control. RPA32 was used as a non-chromatin marker. **(c)** HeLa cells were transfected with the indicated DNA plasmids and lysed in presence of 0,3 mg/ml of RNase A. DNA pull-down of the indicated biotinylated DNA structures, followed by western blot analysis with the indicated antibodies. Please note that the anti-HNRNPD antibody (top panel) recognizes both the wt-HNRNPD endogenous isoforms and the wt-FLAG transfected p45 isoform, whereas the anti-FLAG antibody (lower panel) was used to detect the D and E mutants (which have a lower molecular weight). HNRNPD hybridization (top panel) confirmed the ability of p45 to bind the DNA structure and also served as a pull down normalization control for the D and E mutants. Protein expression levels were not normalized to the amount of effectively pulled-down DNA therefore the higher amount of DNA-bound wt-HNRNPD might be due to both technical or biological reasons. **(d)** Pull-down assay with the biotinylated DNA structures of the human recombinant his-p45 D and E mutants purified from *E. coli.* Western blot assay was performed with the His-tag antibody. Please note that only the E mutant was able to bind the synthetic DNA structure despite its low expression level (as shown in the input lane). Although part of this binding might be non-specific, because it occurs also in the no-DNA control sample, its increase was reproducible in two independent experiments.

Supplementary Figure 2. Analysis of principal DNA end-resection players and checkpoint activation in HeLa HNRNPD -/- cell clones.

(a) HeLa cells were transfected with the siHNRNPD for 24 or 48 hours followed by western blot with the indicated antibody. Lamin A/C was used as a loading control. **(b)** Indicated siRNAs were transfected in HeLa cells for 36 hours followed by incubation 10µM BrdU for additional 16 hours. At the end of incubation, HeLa cells were treated with 1mM of hydroxyurea (HU) for 12 hours followed by cell fix and immunofluorescence with the indicated antibody. Data represent the mean +/- s.d. (n=3 independent experiments). HNRNPD silencing efficacy was assessed by western blot (not shown) and reached approximately 50-60% of protein reduction. *p-value<0,05. For each condition 100 cells

were counted. **(c)** Western blot analysis of HNRNPD protein levels in HeLa ER-AsiSI cells, transfected with the indicated siRNAs, which were used for the DNA end resection assay shown in fig. 3d. 4-OHT: 4-hydroxytamoxifen. **(d)** HeLa cells were transfected with the siCTR or siHNRNPD. *HNRNPD, MRE11, CTIP* and *EXOI* mRNA expression levels were analyzed by real time qRT-PCR and normalized to those of the *GAPDH* gene. (* $p<0.05$; n=3). Error bars denote relatives.d. **(e)** HeLa cells transfected as in (d) were analyzed through the western blot assay with the indicated antibodies**.** GAPDH was used as protein loading control. **(f)** Western blot assay for the MRE11 protein levels upon transfection of HeLa cells with the siRNAs targeting the HNRNPD mRNA. **(g)** HeLa ER-AsiSI were transfected with siCTR or siHNRNPD for 48 hours followed by treatment with 300nM 4-OHT for 1 hour. ChIP analysis was performed by using either an RPA32 antibody or control IgGs (Ab-). RPA32 chromatin binding ability was measured, as a percentage of immunoprecipitated input, from qPCR values of a 200bp amplicon upstream to AsiSI cut site. Data represent the mean +/- s.d. (n=3 independent experiments). **(h)** Western blot analysis of HNRNPD protein levels in HeLa ER-AsiSI transfected with the FLAG-p45 or empty vector, which were used for the ChIP analysis shown in figure 3f.

Supplementary Figure 3. In vitro DNA end-resection assay and HNRNPD/SAF-A reciprocal interaction.

(a) Western blot analysis of MRE11, HNRNPD, SceI-HA expression levels in HeLa pDR-GFP transfected with the indicated siRNAs or plasmids, which were used to calculate %HR frequency as shown in figure 3g. **(b)** HeLa wt and HNRNPD ko cells were treated with 1μM CPT for two hours followed by western blot analysis with the indicated antibodies. Lamin A/C was used as protein loading control. Densitometric analysis of pCHK1 activation was carried out through the ImageJ software. The values of band density corresponding to pCHK1 S345, normalized to the total protein levels, are reported. **(c-d)** HeLa HNRNPD -/- cl10 cells were transfected with the pCEFL-HA HNRNPD isoforms (p45, p40 and p37) with the PAM mutated sequence for 48 hours followed by 1μM CPT for additional two hours. Western blot analysis was performed with the indicated antibodies. Lamin A/C was used as a loading control. Densitometric analysis of pRPA32 S4/8 activation was carried out through the ImageJ software. In a replicate experiment high levels of p37 seemed to inhibit p45-mediated ability to increase phosphoRPA levels. Further investigation of the possible interaction among isoforms is warranted. **(e-g)** Densitometric analysis of the DNA end-resection assay in vitro (shown in figure 4e) with

the nuclear protein extracts from HeLa wt and HNRNPD cl10 cell lines challenged with plasmidic DNA with either 3’, blunt and 5’ overhangs through the ImageJ software.

Supplementary Figure 4. HNRNPD/SAF-A reciprocal interaction.

(a) HeLa cells were transfected with either SAF-A FLAG or HNRNPD-HA, incubated for 48 hours and treated or not for two hours with 1μM CPT. Protein lysates were incubated 30’ on ice with 0,3 μg/ml of RNase A and 50 μg/ml of EtBr followed by centrifugation to remove the debris. Immunoprecipitation was conducted with anti-FLAG antibody O.N. at +4°C. An antibody against total RPA32 was used as a DNA damage control. **(b)** Chromatin enriched purification of HeLa wt and HNRNPD -/- cell lines followed by 1μM CPT treatment for two hours. The western blot analysis was performed with the indicated antibodies; GAPDH and Lamin A/C were used as soluble and insoluble fractions, respectively. Total RPA was used as DNA damage control. Densitometric analysis of SAF-A protein levels was carried out through the ImageJ software. The values of band density corresponding to SAF-A, normalized to the total Lamin A/C levels, are reported.
