# Supplementary material for: Depletion of the RNA binding protein HNRNPD impairs homologous recombination by inhibiting DNA-end resection and inducing R-loop accumulation
Source: Nucleic Acids Res. 2019 Feb 25;47(8):4068–85. doi: 10.1093/nar/gkz076 (PMC6486545; doi:10.1093/nar/gkz076)
Supplement: Supplementary Data [file gkz076_supplemental_files.zip › Supplementary Table 1-.docx]

**Supplementary Table 1: List of primers**

| **Oligo Name** | **Sequence** |
| --- | --- |
| ssDNA bottom 70nt | 5’-TGCAGCTGGCACGACAGGTTTTAATGAATCGGCCAACGCGCGGGGAGAGG CGGTTTGCGTATTGGGCGCT-biotin-3 |
| ssDNA top 21nt | 5’-AGCGCCCAATACGCAAACCGC-3’ |
| ssDNA top 70nt | 5’-AGCGCCCAATACGCAAACCGCCTCTCCCCGCG CGTTGGCCGATTCATTAAAACCTGTCGTGCCAGCTGCA-3’ |
| ssDNA bottom 70nt 5’bio-3’bio | 5’-biotin-TGCAGCTGGCACGACAGGTTTTAATGAATCGGCCAACGCGCGGGGAGAGG CGGTTTGCGTATTGGGCGCT-biotin-3 |
| ssDNA top 70nt 5’bio-3’bio | 5’-biotin-AGCGCCCAATACGCAAACCGCCTCTCCCCGCG CGTTGGCCGATTCATTAAAACCTGTCGTGCCAGCTGCA-biotin-3’ |
| 364 F | 5’-GGGAGACTTGGAGGAGGGAC-3’ |
| 364 R | 5’-GTTCGGTCTGTTCAATCGTCTG-3’ |
| 1754 F | 5-GAAGCCATCCTACTCTTCTCACCT-3’ |
| 1754 R | 5’-GCTGGAGATGATGAAGCCCA-3’ |
| AcrossDBS2 F | 5’-AGCCTCTCCTGCAGCCG-3’ |
| AcrossDBS2 R | 5’-GCGGGAACCAGACCTACCT-3’ |
| No-DBS F | 5’-AGAAGGTGAAGGGAAGCCG-3’ |
| No-DBS R | 5’-ACGCCCTGTATTGAGCACTTT-3’ |
| DSB-F CHIP and DRIP | 5’-TTCCTGCAGCCTCATTTTCT-3’ |
| DSB-R CHIP and DRIP | 5’-TGATGATGCCTTTTCCCTTC-3’ |
| RPA70 F | 5’-AATTCCGGGATATCGATGGTCGGCCAACTGAGCGAG-3’ |
| RPA70 R | 5’-AATTCCGGGGTACCTCACATCAATGCACTTCTCCT-3’ |
| RPA32 F | 5’-ATTAATGGGATCCGATGTGGAACAGTGGATTCGAA-3’ |
| RPA32 R | 5’-AATCTGAGAGCTCGTTATTCTGCATCTGTGGATTT-3’ |
| RPA14 F | 5’-AACTGTCGACAAGGAGATATAAATGGTGGACATGATGGACTTG-3’ |
| RPA14 R | 5’-ATTCAGGGCGGCCGCTCAATCATGTTGCACAATC-3’ |
| RRM1 pFLAG F (mutant A) * | 5’-AATAATGAATTCCATGTTTATAGGAGGCCTTAGC-3’ |
| RRM1 pFLAG R (mutant A) * | 5’-AGGAACGGATCCGGCCCTTTTAGGATCAATCAC-3’ |
| RRM1+CBFNT pFLAG F (mutant B) * | 5’-AATAATGAATTCCATGTCGGAGGAGCAGTTCGGC-3’ |
| RRM1+CBFNTpFLAG R (mutant B) * | 5’-AGGAACGGATCCGGCCCTTTTAGGATCAATCAC-3’ |
| RRM2 pFLAG F (mutant C) | 5’-AATAATGAATTCCAAAATTTTTGTTGGTGGCCTT-3’ |
| RRM2 pFLAG R (mutant C) | 5’-AGGAACGGATCCTTAGGCTACTTTTATTTCACATTT-3’ |
| RRM1+RRM2 pFLAG F (mutant D) | 5’-AATAATGAATTCCATGTTTATAGGAGGCCTTAGC-3’ |
| RRM1+RRM2 pFLAG R (mutant D) | 5’-AGGAACGGATCCTTAGGCTACTTTTATTTCACATTT-3’ |
| RRM2+C-ter pFLAG F (mutant E) | 5’-AATAATGAATTCCAAAATTTTTGTTGGTGGCCTT-3’ |
| RRM2+C-ter pFLAG R (mutant E) | 5’-AAGGACGGATCCTTAGTATGGTTTGTAGCTATTTTG-3’ |
| MRE11 RT-PCR F | 5’-TGCCCAGGAAAATGAAGTG-3’ |
| MRE11 RT-PCR R | 5’-GCCGATCACCCATACAATA-3’ |
| CTIP RT-PCR F | 5’-GCAGCCGTCCTTTCACAGC-3’ |
| CTIP RT-PCR R | 5’-GTCAAATACCGCCTCCGA-3’ |
| EXOI RT-PCR F | 5’-AGAAGAGAAAGACGACAAGCC-3’ |
| EXOI RT-PCR R | 5’-TTCATAGGGAGCCACGAGG-3’ |
| HNRNPD RT-PCR F | 5’-GCCAAAGCCATGAAAACAAAA-3’ |
| HNRNPD RT-PCR R | 5’-GATTCCACCTCACCAAAACCAC-3’ |
| RRM1+RRM2 His F (mutant D) | 5’-AATAATGAATTCCATGTTTATAGGAGGCCTTAGC-3’ |
| RRM1+RRM2 His R (mutant D) | 5’-AGGAACAAGCTTTTAGGCTACTTTTATTTCACATTT-3’ |
| RRM2+C-ter His F (mutant E) | 5’- AATAATGAATTCCAAAATTTTTGTTGGTGGCCTT-3’ |
| RRM2+C-ter His R (mutant E) | 5’-AAGGACGGATCCTTAGTATGGTTTGTAGCTATTTTG-3’ |
| HNRNPD isoforms PAM mutant (G415C) F | 5’-GCACAAAGCCAAAACGCCTTGATCGCCCTGT-3’ |
| HNRNPD isoforms PAM mutant (G415C) F | 5’-ACAGGGCGATCAAGGCGTTTTGGCTTTGTGC-3’ |

* used in the ‘data not shown’ experiments.
